# Care Under Fire: United States Army Physician Survey

**DOI:** 10.7759/cureus.20610

**Published:** 2021-12-22

**Authors:** Clinton R Holaday, Matthew S Holaday

**Affiliations:** 1 Emergency Department, Carle Foundation Hospital, Urbana, USA; 2 Department of Health Sciences, University of Illinois College of Medicine Rockford, Rockford, USA

**Keywords:** military medicine, army medicine, health professions scholarship program, uniformed services university of health sciences, military healthcare, usuhs, hpsp, care under fire, army physician survey, military physician survey

## Abstract

United States Army healthcare has faced increasing criticism in recent years from its own active duty physicians. Internal surveys of active duty physicians demonstrate a lack of successful intervention in an annual exodus countered only by ongoing recruiting efforts. Anecdotal experience suggests much more widespread discontent due to numerous factors than has been reported publicly. This cross-sectional survey study of 94 active duty physicians paints a vivid picture of an organization in crisis: a majority of physicians planning to separate at the end of their obligation, leadership out of touch with the needs of physicians, and systematic deterioration of clinical skills. Subgroup analysis offers insight into why reforms are unlikely to come from within the medical corps. Prospective recruits are also provided the most comprehensive view yet of life as an active-duty Army physician with a majority reporting unmet expectations, a willingness to accept a financial debt in exchange for early separation, and a low likelihood to recommend participating in the current Army medical recruitment programs. Reorientation of recruiting efforts to focus on attending physicians is discussed to address core deficiencies with multiple downstream effects.

## Introduction

The public concept of military medicine inspires the imagination with visions of battlefield valor in dangerous Care Under Fire scenarios when care is first rendered to an injured soldier still in harm’s way. Graduating from medical school without the burden of financial debt builds on this admirable framework, and the very notion of a military officer assumes unrivaled leadership experience. A career as a military physician appears to sell itself, and indeed it does for a number of our nation’s ambitious pre-medical students. Why, then, do the Armed Forces struggle so much with physician retention [1]?

Currently, nearly all military physicians are recruited through one of two training programs: the Health Professions Scholarship Program (HPSP) and the Uniformed Services University of Health Sciences (USUHS). Both offer medical students a blend of full scholarship and living expenses in exchange for an active duty service obligation upon completion of training. Though recruitment mechanisms do exist for fully trained physicians, there are numerous challenges to this method such as direct competition with civilian pay and lifestyle [2].

A 2018 Government Accountability Office study demonstrated that the number of active duty physicians remains well below their authorizations despite the relative success of USUHS and HPSP recruitment efforts [3]. More notably, most physicians will leave active duty as soon as their obligation is met, creating a revolving-door phenomenon of experienced physicians being continually replaced by new graduates [2,4]. Factoring in the blended retirement option suggests further shortfalls can be expected moving forward as the 20-year retirement benchmark loses its attraction [4].

Efforts to define the issues driving physicians out have not been translated into successful intervention. For example, an unpublished 2020 Army Medical Command study of physicians asked nearly identical questions, and produced very similar results, as the published 2018 Army Medical Command study of Army physician satisfaction [5]. While pay differentials between the military and civilian sector receive the most attention because they are easily described and quantifiable, viewing the issue strictly through a financial lens ignores the more challenging task of addressing qualitative job satisfaction and quality of life issues.

Related concerns about critical skill maintenance have attracted attention even beyond military circles. A 2019 US News and World Report survey of active duty surgeons raised serious concerns regarding their employability after the Army due in large part to low case volume [6]. This mirrors ongoing discussions within the emergency medicine community, most recently reflected in a 2020 study by Carius et al discussing very low volumes of emergency procedures performed at military treatment facilities - precisely the type of procedures needed by injured soldiers and severely ill medical patients alike [7].

To date, studies have fallen short of aptly describing the overall military physician experience in a holistic manner and of accurately framing the systemic barriers that hold needed reforms at bay. The logistical challenges in studying this population without the Army’s cooperation must be weighed against expected censorship of any negative results obtained by an Army-approved study. Our civilian-organized cross-sectional survey of active duty physicians was designed with no pre-publication military review and with an emphasis on respondent anonymity. Special focus was on physicians planning to separate at the end of their current obligation as this demographic represents the majority of active duty physicians as graphically demonstrated by Wiesen et al [4]. Sampling all active duty physicians at any given point in time underrepresents this group due to the survivor bias resulting from those separations. This survivor bias also inherently leads to this group’s underrepresentation within senior Army medical leadership. In this study, we looked to directly compare the perspectives of those Army physicians planning to separate with physicians planning to continue on with their Army career. By highlighting these contrasts, our hope is to re-orient the discussion to explain the current lack of reform generated by career Army physician-leaders and prompt movement in a new direction. Furthermore, our study helps lend transparency to those pre-medical students considering accepting an HPSP or USUHS scholarship and thereby obligating themselves more than a decade into the future before they will have treated their first patient. 

## Materials and methods

Military Outlook contacts were searched by job title for keywords matching “physician,” variations of “surgeon,” and common variations of specialties. A total of 702 email addresses of presumed active duty physicians were obtained. Results were screened to include only .mil email addresses and those individuals with rank of Captain or above (the minimum rank for Army physicians). Persons with indications of Reserve status were excluded.

The survey was built using the SurveyMonkey.com platform. Questions were created by the authors for the purposes of this study alone with review and editing by local Institutional Review Board staff of Carle Foundation Hospital. Physicians were contacted twice via email, first with an initial invitation and a follow-up approximately four weeks later. Emails included a weblink to the survey. To maximize anonymity and participation, weblinks were not recipient-specific and respondents were encouraged to share with peers who met inclusion criteria. The survey was open for 60 days and questions included a wide range of topics such as recruitment, quality-of-life, and clinical skills. Mental health assessment questions incorporated a widely-used screening tool, Patient Health Questionnaire-9, for signs of depression [8]. 

In contrast to civilian medicine, most military medical specialties adopt atypical or expanded roles in trauma care in the deployed setting. Therefore, we included questions about all physicians’ clinical trauma skills. However, early in the survey process some respondents provided feedback that they would never have a trauma role. Consequently, we revised the survey and added this response option for relevant clinical skills questions. Due to a low number of responses before this addition and the vast majority of early responses reporting specialties that would be expected to provide trauma care, responses that were provided prior to this added response option were not felt to significantly skew the overall results.

## Results

A total of 106 responses were returned; 94 met inclusion criteria of being either an attending physician or a fellow-level physician currently serving on active duty in the Army. Based on the respondent’s answer to the question “How likely are you to remain on active duty past your current obligation?” the information was then divided into two groups, Separation and Retention. The Separation group was defined as those who responded “Not At All Likely” (67.02%) with the rest comprising the Retention group (32.98%). Chi-Square analysis was performed to determine statistically significant differences between the groups. Regarding Military Medicine Recruitment, only respondents who utilized the HPSP and USUHS programs were included (91 responses total, 60 in the Separation group and 31 in the Retention). Key questions and responses are reported in Tables 1-5. Full survey results can be found within the Appendix [Table 6].

**Table 1:** Demographics HPSP: Health Professions Scholarship Program; USUHS: Uniformed Services University of Health Sciences; ADSO: Active Duty Service Obligation

Demographics	Retention Group (n = 31)	Separation Group (n = 63)
Civilian Medical School Graduate via HPSP	80.65%	74.60%
USUHS Graduate	19.35%	20.63%
Physicians with military experience prior to medical school	41.94% (average 1.5 years)	37.10% (n = 62, average 2.3 years)
Initial ADSO	5.6 years	6.4 years
Years on active duty thus far	11.2 years	10.1 years
Expected years to be served beyond ADSO	11.0 years	1.6 years

**Table 2:** Military Medical Recruitment R = Retention group; S = Separation group

Military Medical Recruitment	Retention Group, R (n = 31) Separation Group, S (n = 60)	Retention Group (R) Separation Group (S)	Retention Group (R) Separation Group (S)	Retention Group (R) Separation Group (S)	Retention Group (R) Separation Group (S)
How much did the financial pressure of physician training (medical school tuition, civilian debt/loan payback, etc.) lead you to join the military? (p = .088)	Overwhelming pressure: R = 9.68%, S = 15.00%	A lot of pressure: R = 29.03%, S = 36.67%	Moderate amount of pressure: R = 12.90%, S = 25.00%	Slight pressure: R = 32.26%, S = 10.00%	Not at all: R = 16.13%, S = 13.33%
When you considered a civilian path to finance medical training at the time of recruitment, how concerning was the prospect of carrying and repaying the debt incurred by medical school loans? (p = .158)	Very concerning: R = 32.26%, S = 41.67%	Moderately concerning: R = 16.13%, S = 25.00%	Somewhat concerning: R = 32.26%, S = 23.33%	Not concerning at all R = 6.45%, S = 8.33%	Would not have required loans: R = 12.9%, S = 1.67%
How likely are you to recommend to a friend or family member to join the Army via the Health Profession Scholarship Program (HPSP)? (p < 0.001)	Very likely: R = 25.81%, S = 3.33%	Likely: R = 41.94%, S = 13.33%	Neither likely nor unlikely: R = 29.03%, S = 15.00%	Unlikely: R = 3.23%, S = 31.67%	Very unlikely: R = 0.00%, S = 36.67%
How likely are you to recommend to a friend or family member to join the Army via the Uniformed Services University of Health Sciences (USUHS)? (p < 0.001)	Very likely: R = 19.35%, S = 1.67%	Likely: R = 32.26%, S = 6.67%	Neither likely nor unlikely: R = 32.26%, S = 21.67%	Unlikely: R = 12.90%, S = 15.00%	Very unlikely: R = 3.23%, S = 55.00%
If an Army physician serving their active-duty service obligation felt a sincere need to separate from the Army before completing their obligation, how likely would you expect the Army to grant a request for early separation? (p = .003)	Very likely: R = 0.00%, S = 1.67%	Likely: R = 6.45%, S = 3.33%	Neither likely nor unlikely: R = 0.00%, S = 0.00%	Unlikely: R = 29.03%, S = 3.33%	Very unlikely: R = 64.52%, S = 91.67%
Army residents generally earn greater pay and benefits than their civilian counterparts. How important is this factor when considering the overall cost-benefit of the HPSP/USUHS program and subsequent active-duty service obligation? (p = .036)	Highly important: R = 22.58%, S = 13.33%	Moderately important: R = 38.71%, S = 16.67%	Not applicable.	Somewhat important: R = 25.81%, S = 48.33%	Not important at all R = 12.9%, S = 21.67%
In general, do you currently feel that an active-duty service obligation is preferable to the average financial obligation that traditional loans impose upon civilians? (p < 0.001)	Active duty service obligation is very preferable: R = 32.26%, S = 5.00%	Active duty service obligation is somewhat preferable: R = 45.16%, S = 21.67%	Neither: R = 16.13%, S = 13.33%	Financial debt is somewhat preferable: R = 6.45%, S = 23.33%	Financial debt is very preferable: R = 0.00%, S = 36.67%
How consistent has your experience as an Army physician been with the expectations that you had at the time of recruitment? (p < 0.001)	Very consistent: R = 19.35%, S = 0.00%	Moderately consistent: R = 41.94%, S = 11.67%	Not applicable.	Somewhat consistent: R = 35.48%, S = 36.67%	Not at all consistent: R = 3.23%, S = 51.67%
If you were offered to trade your current active-duty service obligation for a reasonable financial debt and early separation from the military, how likely would you be to make this trade? (p < 0.001)	Very likely: R = 6.45%, S = 58.33%	Likely: R = 19.35%, S = 23.33%	Neither likely nor unlikely: R = 12.90%, S = 5.00%	Unlikely: R = 29.03%, S = 10.00%	Very unlikely: R = 32.26%, S = 3.33%
If you were offered to trade your current active-duty service obligation for a reasonable financial debt and early transition into the Army Reserves, how likely would you be to make this trade? (p = .309)	Very likely: R = 12.90%, S = 30.00%	Likely: R = 22.58%, S = 26.67%	Neither likely nor unlikely: R = 6.45%, S = 6.67%	Unlikely: R = 25.81%, S = 15.00%	Very unlikely: R = 32.26%, S = 21.67%

**Table 3:** Clinical Skills R = Retention group; S = Separation group

Clinical Skills	Retention Group, R (n = 31) Separation Group, S (n = 63)	Retention Group (R) Separation Group (S)	Retention Group (R) Separation Group (S)	Retention Group (R) Separation Group (S)	Retention Group (R) Separation Group (S)	Retention Group (R) Separation Group (S)
If you deployed today, how would your performance at trauma care on day one compare to a civilian physician of the same specialty who is based in a reasonably busy trauma center? (p = .035)	Perform much better: R = 9.68%, S = 4.76%	Perform better: R = 32.26%, S = 9.52%	Perform at a similar level: R = 25.81%, S = 26.98%	Perform worse: R = 9.68%, S = 28.57%	Perform much worse: R = 12.90%, S = 23.81%	I would not treat trauma patients: R = 9.68%, S = 6.35%
If a typical military physician were deployed today, how would you expect that military physician's trauma care on day one of deployment to compare to a civilian physician of the same specialty based in a reasonably busy trauma center? (p = .016)	Perform much better: R = 10.34%, S = 6.67%	Perform better: R = 34.48%, S = 8.33%	Perform at a similar level: R = 17.24%, S = 25.00%	Perform worse: R = 31.03%, S = 35.00%	Perform much worse: 6.90%, S = 25.00%	Not applicable.
If you deployed today, how would the Army's impact on your skill maintenance affect outcomes of wounded soldiers? (p = .085)	My Army positions have further developed my skills and greatly decreased the risk of harm: R = 3.45%, S = 6.78%	My Army positions have further developed my skills and decreased the risk of harm: R = 3.45%, S = 5.08%	My Army positions have maintained my skills without any change in expected outcomes: R = 34.48%, S = 22.03%	My Army positions have deteriorated my skills and increased the risk of harm: R = 58.62%, S = 45.76%	My Army positions have deteriorated my skills and greatly increased the risk of harm: R = 0.00%, S = 20.34%	Not applicable.
How would you rate your clinical skills as they relate to trauma patients against a civilian physician in your specialty? (p < 0.001)	My skills are much stronger: R = 6.90%, S = 10.00%	My skills are stronger: R = 51.72%, S = 8.33%	Our skills are similar or equal: R = 24.14%, S = 38.33%	My skills are weaker: R = 6.90%, S = 28.33%	My skills are much weaker: R = 10.34%, S = 15.00%	Not applicable.
What impact have your Army positions had on your ability to treat complex trauma patients? (p = .014)	Very positive impact: R = 10.34%, S = 3.33%	Positive impact: R = 34.48%, S = 10.00%	Little to no impact either way: R = 31.03%, S = 33.33%	Negative impact: R = 17.24%, S = 30.00%	Very negative impact: R = 6.90%, S = 23.33%	Not applicable.
What impact have your Army positions had on your ability to treat complex medical patients? (p = .110)	Very positive impact: R = 3.33%, S = 4.76%	Positive impact: R = 20.00%, S = 9.52%	Little to no impact either way: R = 26.67%, S = 12.70%	Negative impact: R = 36.67%, S = 38.10%	Very negative impact: R = 13.33%, S = 34.92%	Not applicable.
Compared to a number that you feel would be adequate to maintain your desired clinical skills, how would you describe the number of opportunities to perform the relevant procedures in your Army duty positions? Please disregard continuing medical education and off-duty employment extraneous to reserved training time. (p = .184)	Many more opportunities: R = 3.33%, S = 1.59%	More opportunities: R = 16.67%, S = 3.17%	Just enough opportunities: R = 13.33%, S = 12.70%	Fewer opportunities: R = 30.00%, S = 30.16%	Far fewer opportunities: R = 36.67%, S = 46.03%	I do not perform procedures: R = 0.00%, S = 6.35%
Compared to a number that you feel would be adequate to maintain your desired clinical skills, how would you describe the number of opportunities to perform relevant patient care in your Army duty positions? Please disregard continuing medical education and off-duty employment extraneous to reserved training time. (p = .188)	Many more opportunities: R = 3.33%, S = 3.17%	More opportunities: R = 16.67%, S = 4.76%	Just enough opportunities: R = 30.00%, S = 26.98%	Fewer opportunities: R = 36.67%, S = 33.33%	Far fewer opportunities: R = 13.33%, S = 31.75%	Not applicable.

**Table 4:** Command Climate R = Retention group; S = Separation group

Command Climate	Retention Group, R (n = 31) Separation Group, S (n = 63)	Retention Group (R) Separation Group (S)	Retention Group (R) Separation Group (S)	Retention Group (R) Separation Group (S)	Retention Group (R) Separation Group (S)
To what extent does local leadership understand the benefit of continuing medical education and/or off-duty employment that you may need to maintain your skills? (p < 0.001)	Shows strong understanding: R = 26.67, S = 1.59%	Shows moderate understanding: R = 30.00%, S = 14.29%	Not applicable.	Shows somewhat of an understanding: R = 26.67%, S = 34.92%	Shows little to no understanding: R = 16.67%, S = 49.21%
To what extent does senior leadership understand the benefit of continuing medical education and/or off-duty employment that you may need to maintain your skills? (p < 0.001)	Shows strong understanding: R = 13.33%, S = 1.59%	Shows moderate understanding: R = 33.33%, S = 4.76%	Not applicable.	Shows somewhat of an understanding: R = 23.33%, S = 23.81%	Shows little to no understanding: R = 30.00%, S = 69.84%
What role does local leadership play in your pursuit of continuing medical education and/or off-duty employment that you may need to maintain your skills? (p = .001)	Leadership strongly supports: R = 13.33%, S = 3.17%	Leadership supports: R = 50.00%, S = 19.05%	Leadership neither supports nor discourages: R = 26.67%, S = 41.27%	Leadership discourages: R = 10.00%, S = 17.46%	Leadership strongly discourages: R = 0.00%, S = 19.05%
What role does senior leadership play in your pursuit of continuing medical education and/or off-duty employment that you may need to maintain your skills? (p = .003)	Leadership strongly supports: R = 6.67%, S = 0.00%	Leadership supports: R = 30.00%, S = 9.52%	Leadership neither supports nor discourages: R = 43.33%, S = 49.21%	Leadership discourages: R = 20.00%, S = 20.63%	Leadership strongly discourages: R = 0.00%, S = 20.63%
Senior Army medical leadership positions are held by individuals who are promoted and assigned to positions based on their leadership skills and administrative training. (p = .006)	Strongly agree: R = 6.45%, S = 3.17%	Agree: R = 29.03%, S = 7.94%	Neither agree nor disagree: R = 25.81%, S = 20.63%	Disagree: R = 32.26%, S = 30.16%	Strongly disagree: R = 6.45%, S = 38.10%
How would you compare Army medical leaders to their corresponding civilian counterparts in leadership and administrative guidance? (p = .002)	Army medical leaders provide far superior leadership and administrative guidance: R = 6.67%, S = 0.00%	Army medical leaders provide superior leadership and administrative guidance: R = 10.00%, S = 0.00%	Army medical leaders provide equivalent leadership and administrative guidance: R = 36.67%, S = 34.92%	Army medical leaders provide inferior leadership and administrative guidance: R = 46.67%, S = 42.86%	Army medical leaders provide far inferior leadership and administrative guidance: R = 0.00%, S = 22.22%
How strong of an understanding do you feel Army medical leadership possesses regarding the needs of active duty physicians? (p < 0.001)	Strong understanding: R = 3.23%, S = 0.00%	Moderate understanding: R = 35.48%, S = 4.76%	Not applicable.	Weak understanding: R = 54.84%, S = 50.79%	Little to no understanding: R = 6.45%, S = 44.44%
How would you rate the performance of Army medical leadership in advancing the clinical skills of active duty physicians? (p < 0.001)	Army medical leadership performs very well: R = 0.00%, S = 0.00%	Army medical leadership performs well: R = 6.45%, S = 4.76%	Army medical leadership performs adequately: R = 25.81%, S = 15.87%	Army medical leadership performs poorly: R = 61.29%, S = 28.57%	Army medical leadership performs very poorly: R = 6.45%, S = 50.79%
How would you rate the performance of Army medical leadership in advancing the deployment readiness of active duty physicians? (p = .005)	Army medical leadership performs very well: R = 0.00%, S = 0.00%	Army medical leadership performs well: R = 16.13%, S = 6.35%	Army medical leadership performs adequately: R = 48.39%, S = 23.81%	Army medical leadership performs poorly: R = 29.03%, S = 34.92%	Army medical leadership performs very poorly: R = 6.45%, S = 34.92%
How would you rate the performance of Army medical leadership in advancing the job satisfaction and wellness of active duty physicians? (p < 0.001)	Army medical leadership performs very well: R = 0.00%, S = 0.00%	Army medical leadership performs well: R = 3.23%, S = 0.00%	Army medical leadership performs adequately: R = 16.13%, S = 6.35%	Army medical leadership performs poorly: R = 58.06%, S = 25.40%	Army medical leadership performs very poorly: R = 22.58%, S = 68.25%
In general, how do you feel Army medical leaders encourage or discourage efforts by physicians to improve Army healthcare? (p = .007)	Strongly encourages and/or facilitates: R = 3.23%, S = 0.00%	Encourages and/or facilitates: R = 22.58%, S = 4.76%	Neither encourages nor discourages: R = 38.71%, S = 28.57%	Discourages and/or hinders: R = 32.26%, S = 46.03%	Strongly discourages and/or hinders: R = 3.23%, S = 20.63%
To what extent do you feel encouraged to raise any concerns to your leadership? (p = .054)	Very encouraged: R = 3.23%, S = 3.17%	Encouraged: R = 32.26%, S = 9.52%	Neither encouraged nor discouraged: R = 32.26%, S = 33.33%	Discouraged: R = 25.81%, S = 33.33%	Very discouraged: R = 6.45%, S = 20.63%
To what extent do you feel encouraged to offer solutions to your leadership? (p = .011)	Very encouraged: R = 3.23%, S = 3.17%	Encouraged: R = 35.48%, S = 11.11%	Neither encouraged nor discouraged: R = 35.48%, S = 31.75%	Discouraged: R = 25.81%, S = 34.92%	Very discouraged: R = 0.00%, S = 19.05%
How likely is it that the concerns you raise will be adequately addressed? (p = .002)	Very likely to be adequately addressed: R = 0.00%, S = 1.59%	Likely to be adequately addressed: R = 9.68%, S = 3.17%	Neither likely nor unlikely to be adequately addressed: R = 38.71%, S = 9.52%	Unlikely to be adequately addressed: R = 35.48%, S = 38.10%	Very unlikely to be adequately addressed: R = 16.13%, S = 47.62%
How empowered do you feel to enact change in your organization? (p < 0.001)	Very empowered: R = 3.23%, S = 3.17%	Empowered: R = 25.81%, S = 7.94%	Not applicable.	Somewhat empowered: R = 48.39%, S = 19.05%	Do not feel empowered to enact any change: R = 22.58%, S = 69.84%
What is your assessment of the personal risk of retribution due to speaking out against decisions and policies that you disagree with? (p = .021)	Significant personal risk: R = 6.45%, S = 30.16%	Moderate personal risk: R = 29.03%, S = 12.70%	Not applicable.	Slight personal risk: R = 41.94%, S = 28.57%	Little to no risk: R = 22.58%, S = 28.57%

**Table 5:** Proposed reform R = Retention group; S = Separation group

Proposed Reform	Retention Group, R (n = 31) Separation Group, S (n = 63)	Retention Group (R) Separation Group (S)	Retention Group (R) Separation Group (S)	Retention Group (R) Separation Group (S)	Retention Group (R) Separation Group (S)
In general, the reliable replacement of physicians leaving active duty by new graduates with active-duty service obligations encourages leadership to work to improve the active-duty physician experience. (p = .016)	Strongly agree: R = 3.33%, S = 0.00%	Agree: R = 6.67%, S = 4.76%	Neither agree nor disagree: R = 16.67%, S = 9.52%	Disagree: R = 56.67%, S = 33.33%	Strongly disagree: R = 16.67%, S = 52.38%
In general, the reliable replacement of physicians leaving active duty by new graduates with active-duty service obligations discourages leadership from working to improve the active-duty physician experience. (p = .015)	Strongly agree: R = 13.33%, S = 49.21%	Agree: R = 60.00%, S = 30.16%	Neither agree nor disagree: R = 20.00%, S = 14.29%	Disagree: R = 3.33%, S = 4.76%	Strongly disagree: R = 3.33%, S = 1.59%
In general, limiting recruitment to attending physicians instead of aspiring medical students would most likely encourage leadership to work to improve the active-duty physician experience. (p = .460)	Strongly agree: R = 16.67%, S = 26.98%	Agree: R = 33.33%, S = 39.68%	Neither agree nor disagree: R = 30.00%, S = 22.22%	Disagree: R = 6.67%, S = 6.35%	Strongly disagree: R = 13.33%, S = 4.76%
In general, limiting recruitment to attending physicians instead of aspiring medical students would most likely discourage leadership from working to improve the active-duty physician experience. (p = .542)	Strongly agree: R = 3.33%, S = 1.61%	Agree: R = 0.00%, S = 3.23%	Neither agree nor disagree: R = 46.67%, S = 33.87%	Disagree: R = 33.33%, S = 33.87%	Strongly disagree: R = 16.67%, S = 27.42%

Ninety-one of 94 respondents participated in the HPSP or USUHS program. Of these 91 respondents as displayed in Table 1, 86% reported some degree of financial pressure led them to join the military, and 87% felt concerned by the prospect of carrying and repaying medical school debt. There was no statistical significance between groups on this matter, but other recruitment-related topics yielded significant differences. 70% of the Separation group felt they would be unlikely or very unlikely to recommend the HPSP program to a friend or family member, and 93% of this group reported they would be very unlikely to recommend the USUHS program. In contrast, a majority of the Retention group would recommend both programs. Ninety-two percent of the Separation group compared to 65% of the Retention group felt it would be very unlikely that an Army physician in sincere need would be allowed to separate from the military before their obligation was complete. Only 30% of the Separation group compared to 61% of the Retention group felt that better pay and benefits during medical training should be considered a very or moderately important factor in the overall cost-benefit assessment of the HPSP and USUHS programs. Sixty percent of the Separation group felt that a financial debt is somewhat or very preferable to an active duty service obligation, though 77% of the Retention group reported that an active duty service obligation is somewhat or very preferable. While 19% and 42% of the Retention group felt their experience has been very or moderately consistent, respectively, with their expectations at the time of recruitment, 0% and 12% of the Separation group felt their experience has been very or moderately consistent, respectively, with their expectations. While all respondents in the Separation group reported planning to separate at the end of their current obligation, 58% reported that they would be very likely and 23% would be likely to accept a reasonable financial debt for earlier separation. Fifty-seven percent of the Separation group would also be willing to accept a reasonable financial debt in exchange for early transfer into the Army Reserves, though this did not reach statistical significance between groups [Table 1]. 

Respondents also gave candid free-response answers regarding their advice to those who may be considering an HPSP or USUHS scholarship which are reported within the Appendix of this publication [Table 6].

Fifty-two percent of the Separation group who would be expected to treat trauma patients on deployment felt their personal performance at trauma care on day one would be worse or much worse than a civilian peer based in a trauma center, while only 23% of the Retention group reported similar feelings. The Separation group had similarly negative views about the typical military physician’s trauma skills as 60% felt the care rendered would be worse or much worse. The Retention group has nearly evenly split in their opinions regarding typical military physicians’ trauma care. When respondents were asked to rate their clinical skills as they relate to trauma patients against a civilian physician in their specialty in general, significant differences were found again between groups with similar opposing trends [Table 2].

There was no significant difference between groups when asked about how the Army’s impact on their skill maintenance would affect the outcomes of wounded soldiers if deployed today. However, both groups clearly trend toward physicians’ Army positions causing a deterioration of critical trauma skills and increased risk of harm to wounded soldiers [Table 2]. While there was a significant difference between the groups when asked about the impact of respondents’ Army positions on their ability to treat complex trauma patients, there was no significant difference when questioned about complex medical patients with clear trends in both groups indicating their Army positions have had a negative or very negative impact on their ability [Table 2].

No difference was identified when respondents were asked to compare the number of opportunities to perform relevant procedures and patient care to the number needed to maintain their desired clinical skills. Forty-three percent and 30% of all respondents felt they received far fewer or fewer procedure opportunities than would be adequate, respectively. Twenty-six percent and 34% felt they received far fewer or fewer patient care opportunities with 28% reporting just enough opportunities [Table 5].

Sixty-eight percent of the Separation group disagreed or strongly disagreed that senior Army medical leadership positions are held by individuals who are promoted and assigned to positions based on their leadership skills and administrative training with 21% neither agreeing or disagreeing [Table 3]. The Retention group offered no clear trend on this matter though the difference between groups was significant. Similarly, 65% of the Separation group felt Army medical leaders provide inferior or far inferior leadership and administrative guidance compared to corresponding civilian counterparts, and no respondents in the Separation group felt Army medical leaders provided superior leadership. While 37% of the Retention group felt Army medical leaders provided equivalent leadership and administrative guidance, 47% felt it to be inferior to corresponding civilian counterparts. Both groups gave poor marks to the role of Army medical leadership in identifying the needs of and advancing the interest of active duty physicians, including deployment readiness, with the Separation group offering significantly worse marks across these inquiries [Table 3].

Sixty-seven percent of the Separation group felt that Army medical leaders discourage or strongly discourage efforts by physicians to improve Army healthcare, while no clear trend was identified in the Retention subgroup [Table 3]. No statistical difference was identified between groups with regards to raising concerns to leadership, though data trended toward the Separation group feeling discouraged or very discouraged from doing so (54%) as opposed to encouraged or very encouraged (13%). The Retention group offered no clear trend on this matter. Significance was met on perceptions of the likelihood that concerns will be adequately addressed. Thirty-eight percent and 48% of the Separation group felt their concerns would be unlikely or very unlikely to be adequately addressed, respectively, compared to 35% and 16% of the Retention group, with 39% of the Retention group reporting their concerns would be neither likely nor unlikely to be adequately addressed. Seventy percent of the Separation group felt they did not feel empowered at all to enact change in their organization compared to 23% of the Retention group, though only two respondents in the Separation group and one respondent in the Retention group felt very empowered. Finally, the Separation group reported feeling significantly more personal risk of retribution due to speaking out against policies and decisions that they disagree with than the Retention group. Only 29% of the Separation group and 23% of the Retention group felt little to no risk, but 30% of the Separation subgroup felt significant personal risk compared to 6% of the Retention subgroup [Table 3]. Respondents from both groups offered similar rates of concern over different specific forms of retribution [Table 5].

Significant differences were found throughout inquiries probing physicians' assessments of local and senior leadership’s valuing and supporting the pursuit of continuing medical education and/or off-duty employment in maintaining skills. While 49% and 70% of the Separation group felt local and senior leadership, respectively, show little to no understanding, no clear trend was identified in the Retention group. The Separation group was mixed on their opinions of the role local leadership plays in these pursuits, but 42% felt that senior leadership discourages or strongly discourages these pursuits, with 49% feeling that senior leadership neither supports nor discourages. Conversely, 63% of the Retention group felt local leadership supports or strongly supports these pursuits, though this trend was much less pronounced regarding senior leadership’s role [Table 3]. 

Thirty-three percent and 52% of the Separation group disagreed or strongly disagreed, respectively, that the reliable replacement of physicians leaving active duty by new graduates with active duty service obligations encourages leadership to work to improve the active duty physician experience [Table 4]. While 57% and 17% of the Retention group also disagreed or strongly disagreed, respectively, this difference met significance. This result was reinforced by the question being inverted: 30% and 49% of the Separation group agreed or strongly agreed, respectively, that this mechanism discourages leadership from working toward improvement. While 60% and 13% of the Retention subgroup agreed or strongly agreed as well, this difference also met significance [Table 4]. Differences were not identified when respondents were queried regarding their expectations of the impact on limiting recruitment to attending physicians instead of aspiring medical students. Of all respondents, 24% strongly agreed and 38% agreed that this change would likely encourage leadership to work to improve the active-duty physician experience, with only 16% disagreeing or strongly disagreeing [Table 5]. Only four respondents indicated that limiting recruitment to attending physicians would discourage improvement [Table 4].

## Discussion

Results of this survey study describe an organization struggling to meet the needs of its physicians across a broad spectrum of issues. Breaking responses into Separation and Retention groups reveals a striking dichotomy between the physicians who plan to separate at the end of their obligation and those who will remain in the military and offers a unique perspective compared to prior studies of military physicians [2,4,5]. The active duty service obligation functioned as an unintended screening tool for those with an approving view of the Army physician experience. These physicians are not only more likely to recommend the HPSP/USUHS program but also to view more positively their own clinical skills and those of the average military physician [Tables 2,3]. Additionally, those opting to stay felt more comfortable taking concerns to leadership and felt significantly more positive about Army medical leadership's understanding and handling of physician issues across numerous inquiries [Table 4]. Due to the heavy attrition rate and the nature of physician promotion within the medical corps being based primarily on time-in-service, one can reasonably expect the Retention group represents physicians who will be or are in senior leadership positions. This selection bias may help explain the disconnect that exists between leadership and the physicians represented by the Separation group.

For example, the HPSP/USUHS programs are generally seen as successful, though internal approval varies. The Retention group views these programs favorably and will likely carry these positive opinions into leadership positions that will, in turn, reinforce these same recruiting methods [Table 2]. Unfortunately, survey results indicate the majority of physicians recruited in this manner disapprove of the programs [Table 2,6]. While both groups reported financial pressure and concern over debt were key factors in their decision to join the Army, significantly more of the Separation group felt their expectations were not met and most of this group felt a financial debt would be preferable to the lengthy service obligation. Over 75% of the Separation group even reported they would be willing to accept financial debt in exchange for early separation and would also be willing to accept a transition into the Army Reserves [Table 2]. This marked disconnect should raise alarms. From the vantage point of the Separation group, these recruiting programs share more overlap with a predatory loan than with a benign and beneficial scholarship program.

Even more disconcerting is the reported shortfall in clinical abilities. Physicians in the Separation group believed their clinical trauma skills to be inferior to civilian physicians, and even the Retention group respondents voiced concerns about their trauma training [Table 3]. Placing physicians in administrative roles or in low-acuity medical treatment facilities without the opportunity for ongoing clinical trauma experience, a common practice in Army medicine evidenced by 22.6% of physician respondents in the 2018 MEDCOM survey reporting majority-administrative duties, can only result in skill regression [5]. Expecting physicians to balance the practiced trauma skills needed on deployment, the routine care and health maintenance needed by Army units in garrison, and the typical clinical skills required for physicians to transition into civilian practice may be viewed by some as reflective of a versatile medical corps that meets the needs of the Army. To many who answered this survey and others living in a modern medical world of specialization, this more likely reflects inferiority in one or more of those medical niches as physicians are placed in roles that are misaligned with their level and area of training. This sentiment is reflected in our survey results where 64% of all respondents reported increased risk of harm to wounded soldiers due to the Army’s negative impact on clinical skill maintenance [Table 3]. For these reasons and others, physicians who do successfully transition from military to civilian practice more often do so in spite of, rather than because of, their Army experience.

Results also support criticism of the institution as being recalcitrant to change. Command climate responses describe a generally suppressive atmosphere that stifles physicians from attempting to improve the system, with perceived threats of retribution and an overwhelming air of futility expressed by the Separation group in particular [Table 4]. Acceptance of the status quo then becomes a de facto requirement for career advancement which, over time, results in the next generation of leadership continuing the same policies that are driving high attrition rates. In aggregate, the command climate inquiries of this study reflect a resounding vote of no-confidence in Army medical leadership’s ability to maintain physician skills and improve overall physician satisfaction [Table 4]. 

There are limitations and challenges of studying an active-duty military population that must be acknowledged. Several senior Army medical officers were contacted with a request for a comprehensive list of physicians that would meet inclusion criteria. Human Resources Command was also contacted in an attempt to obtain more objective demographic data related to recruitment and retention. These requests were declined or ignored. The imperfect method used in lieu of an email listserv resulted in many email invitations failing to be received (though it is unclear how many). Anecdotally, a decline in an individual’s motivation is typically accompanied by less diligent Army Outlook compliance and likely served to decrease participation by many of the more disenfranchised physicians. Furthermore, the cumbersome process of accessing Army Outlook accounts is typically done on government computers which offer warnings that users’ communication may be monitored. This likely added to apprehension among participants and limited both data analysis and participation. For example, several respondents declined to provide their specialty with one response explicitly stating, “I would prefer to not provide this information as I fear reprisal for being honest about the quality of army medicine.” However, allowing respondents to share the survey invitation may have introduced a snowball effect of like-minded individuals. Still, we feel the breakdown in Retention and Separation group numbers are reasonably consistent with Army physicians overall and support the results. The Army could repeat this study with the entire listserv which may clarify certain results and produce more reliable trends, especially within the smaller Retention group.

Appropriate analysis of the mental health components of this survey remains planned but beyond the scope of this report.

## Conclusions

We authors suggest the results of the survey support our premise that the failure of meaningful reform stems from inherent, organizational resistance to change and a leadership is simply out of touch with the majority of Army physicians. Our proposed change of eliminating the HPSP and USUHS programs deserves strong consideration for its relative simplicity to enact by civilian overseers and expected multidimensional benefits. From an ethical standpoint, these programs are questionable at best, legally obligating recruits to up to 14 years of active duty service with, as these results demonstrate, frequently unmet expectations. With a majority of all respondents willing to accept financial debt in exchange for early separation and a lack of clear evidence that these programs produce superior military physicians, their greatest strength appears to be locking pre-medical students into long obligations. On the other hand, re-focusing recruitment on attending physicians may increase retention and satisfaction as attendings are better equipped to make better informed decisions with less financial pressure, leading to more accurate expectations of their commitment. This would finally provide direct feedback from prospective physician-recruits (as opposed to pre-medical students) and accountability to Army medical leadership if and when recruiting goals are not met. It is important to note that the HPSP/USUHS recruitment pipeline is years-long and even sudden elimination would not be felt at the clinical level for at least four years; longer if the effect of fewer resident physicians at medical centers are discounted. Other solutions such as shifting to a largely Reserve force warrant consideration if the military cannot guarantee sufficient clinical skill maintenance while physicians serve on active duty.

In the meantime, potential recruits should be aware of the discrepancies between physicians planning to remain in the military and those planning to leave, and they should seek out references from both. To be clear, neither this data nor anecdotal experience suggests any recruitment effort is malignant or underhanded. The Retention responses demonstrate that many physicians genuinely believe the HPSP and USUHS programs to be excellent choices while the Separation group sees these programs as helping reinforce a broken system that erodes the very clinical skills it pays for.

Every year, a truly exceptional group of Americans accept the many unknowns and risk of military service to provide care to our nation’s soldiers. The concerning narrative that this survey supports may well be one of the most unexpected realizations during their time in service: that the quality of this care is under fire by the very system it serves. Due to the combination of well-intended but misguided recruiting practices, a self-reinforcing mechanism of leadership selection, and the longstanding administrative negligence regarding clinical skill maintenance described here, it is unlikely that sustainable and meaningful reform will come from within the organization. We believe significant transformation can only be achieved through civilian oversight and concrete action should be taken as swiftly as possible.

## Appendices

**Table 6:** Full Survey Results

Army Physician Survey				
Q1. How much did the financial pressure of physician training (medical school tuition, civilian debt/loan payback, etc) lead you to join the military?				
Answer Choices	Responses			
I felt an overwhelming amount of financial pressure	12.77%	12		
I felt a lot of financial pressure	32.98%	31		
I felt a moderate amount of financial pressure	21.28%	20		
I felt slight financial pressure	17.02%	16		
Not at all	15.96%	15		
	Answered	94		
	Skipped	0		
Q2. When you considered a civilian path to finance medical training at the time of recruitment, how concerning was the prospect of carrying and repaying the debt incurred by medical school loans?				
Answer Choices	Responses			
Very concerning	37.23%	35		
Moderately concerning	22.34%	21		
Somewhat concerning	26.60%	25		
Not concerning at all	8.51%	8		
I would not have required loans to pay for medical training as a civilian.	5.32%	5		
	Answered	94		
	Skipped	0		
Q3. How likely are you to recommend to a friend or family member to join the Army via the Health Profession Scholarship Program (HPSP)?				
Answer Choices	Responses			
Very likely	10.64%	10		
Likely	23.40%	22		
Neither likely nor unlikely	19.15%	18		
Unlikely	23.40%	22		
Very unlikely	23.40%	22		
	Answered	94		
	Skipped	0		
Q4. How likely are you to recommend to a friend or family member to join the Army via the Uniformed Services University of Health Sciences (USUHS)?				
Answer Choices	Responses			
Very likely	7.45%	7		
Likely	15.96%	15		
Neither likely nor unlikely	24.47%	23		
Unlikely	14.89%	14		
Very unlikely	37.23%	35		
	Answered	94		
	Skipped	0		
Q5. If an Army physician serving their active duty service obligation felt a sincere need to separate from the Army before completing their obligation, how likely would you expect the Army to grant a request for early separation?				
Answer Choices	Responses			
Very likely	1.06%	1		
Likely	4.26%	4		
Neither likely nor unlikely	0.00%	0		
Unlikely	12.77%	12		
Very unlikely	81.91%	77		
	Answered	94		
	Skipped	0		
Q6. Army residents generally earn greater pay and benefits than their civilian counterparts. How important is this factor when considering the overall cost-benefit of the HPSP/USUHS program and subsequentactive duty service obligation?				
Answer Choices	Responses			
Highly important	15.96%	15		
Moderately important	23.40%	22		
Somewhat important	40.43%	38		
Not important at all	20.21%	19		
	Answered	94		
	Skipped	0		
Q7. In general, do you currently feel that an active duty service obligation is preferable to the average financial obligation that traditional loans impose upon civilians?				
Answer Choices	Responses			
An active duty service obligation is very preferable to financial debt.	13.83%	13		
An active duty service obligation is somewhat preferable to financial debt.	28.72%	27		
Neither an active duty service obligation nor a financial debt is generally preferable over the other.	14.89%	14		
A financial debt is somewhat preferable to an active duty service obligation.	18.09%	17		
A financial debt is very preferable to an active duty service obligation.	24.47%	23		
	Answered	94		
	Skipped	0		
Q8. How consistent has your experience as an Army physician been with the expectations that you had at the time of recruitment?				
Answer Choices	Responses			
Very consistent	6.38%	6		
Moderately consistent	21.28%	20		
Somewhat consistent	37.23%	35		
Not at all consistent	35.11%	33		
	Answered	94		
	Skipped	0		
Q9. If you were offered to trade your current active duty service obligation for a reasonable financial debt and early separation from the military, how likely would you be to make this trade?				
Answer Choices	Responses			
Very likely	40.43%	38		
Likely	23.40%	22		
Neither likely nor unlikely	7.45%	7		
Unlikely	15.96%	15		
Very unlikely	12.77%	12		
	Answered	94		
	Skipped	0		
Q10. If you were offered to trade your current active duty service obligation for a reasonable financial debt and early transition into the Army Reserves, how likely would you be to make this trade?				
Answer Choices	Responses			
Very likely	23.40%	22		
Likely	24.47%	23		
Neither likely nor unlikely	6.38%	6		
Unlikely	20.21%	19		
Very unlikely	25.53%	24		
	Answered	94		
	Skipped	0		
Q11. What advice would you give to someone considering joining the Army in order to pay for medical school? Please avoid sharing personal identifying information.				
Answered	74			
Skipped	20			
Respondents	Response Date	Responses	Tags	
1	Oct 26 2020 08:22 AM	I would recommend not making the decisions a financial one. The military requires addition hardships and if you join the military with out a willingness to be apart of something bigger or with out a willingness nto make personal sacrifice it would not be worth it. The financial gains of a military scholarship is nice but the financial gain does not cover the personal sacrifice the military demands		
2	Oct 12 2020 05:34 PM	Make sure you are doing it for the right reasons - to actually serve, even for a short period of time. It isn't entirely about the money (or shouldn't be). If it is, you will likely be unhappy.		
3	Oct 09 2020 04:11 AM	absolutely do not join the army. we are under-paid, under-supported, and over-worked. i saw a memorandum from the year 1999 in which the then-consultant from my specialty voiced these concerns, and nothing has changed in more than 20 years. i do not expect anything eill ever change. military medicine is dying, the only people that stay are academy/usuhs graduates who owe in some cases more than 14 years of obligation. the debt you incur from a civilian education is easily re-payable once you enter civilain practice. and finally, leadership is rife with incompetence. all you need to do to get promoted is stick around long enough and "suck-up" the the right people. this ultimately means that non-medical personnel will be in charge of you. nurses and administrators are not able to adequately determine what physicians needs in order to practice their craft.		
4	Oct 07 2020 01:48 PM	Only do it if the financial obligation of civilian school is overwhelming. And realize that military medicine is constantly changing and you may not get to do what you wish to.		
5	Oct 01 2020 03:31 PM	Research the residency programs available, the location of the residency programs, and locations of future duty stations for your chosen specialty		
6	Oct 01 2020 03:04 PM	They need to know what they're getting into. It shouldn't be for financial reasons at all, because it will even out in the end as civilian programs will help forgive your debt. And debt may be preferable to moving every 3 years, and being sent overseas at the drop of a hat.		
7	Oct 01 2020 10:11 AM	Just don't! Debt is better than complete lack of control over your life, lack of respect, constantly being treated like an uneducated 18 year old, and unfair financial compensation.		
8	Sep 30 2020 08:27 PM	the GME training is great but there is way too much uncertainty once you become an attending - joining the Army to may for medical school is not worth it. especially as you can't quit a job even if you find yourself in a toxic work place with incompetent leadership. also the lowest ranking people in a medcen or meddac are the ones that are tasked to go on ridiculous trips (TDYs) to the middle of nowhere to backfill as urgent care docs.		
9	Sep 30 2020 06:31 PM	I would advise them to be sure that they understand they are signing up to join the Army and that they will be expected to support the Army's mission first. This may mean that they have to learn to practice outside of their particular training specialty. Additionally, they will be expected to represent the Medical Corps as officers in the Army and should make every effort to learn the Army's processes and procedures.		
10	Sep 30 2020 01:13 PM	That it might not be as advantageous from a financial standpoint if you are doing a surgical subspeciality or longer residency program (longer than 4 years) that incurs a longer committment/obligation.		
11	Sep 30 2020 12:11 PM	Do the math based on your anticipated post-residency income. If you anticipate being a high earner, the HPSP is not worth it as you will be able to quickly pay off your loans. However, if you will be a low earner, then HPSP will be worth it.		
12	Sep 30 2020 11:46 AM	Do not sign up for Army medicine. The Army does not value physicians as professionals. They will treat you like children and try to force you into an admin role. Do med school and residency in the civilian sector so you have control. Then, sign up to come into the Army for three years to serve you country and get loan repayments. They will pay you very poorly and misuse your schools for years if you have a service obligation. Just don't do it.		
13	Sep 30 2020 11:09 AM	If you are doing it for financial reasons, strongly reconsider the Army as a preferable alternative.		
14	Sep 30 2020 10:58 AM	Only do it if you truly would like to serve		
15	Sep 30 2020 09:21 AM	No free lunch when it comes to medical school. Time or money. Potentially the longest indentured servitude that still exists in the United States. The value of freedom to decide your future should not be overlooked.		
16	Sep 30 2020 09:10 AM	Financial considerations should be secondary. Join the Armed Forces if you feel duty to serve your country.		
17	Sep 29 2020 09:37 PM	Think very hard before doing it if being strictly clinical is what you love as it is very difficult to avoid useless / boring admin positions and avoid losing your clinical skills despite the army saying otherwise for all specialties. rather than pushing all specialites to do full time clinic / OR etc, they push everyone into administrative positions that few actually desire which is the biggest reason >85% of all specialities get out after the initial ADSO. If they just let docs be docs and pushed them to maintain their skills (even if it meant allowing them to moonlight more to get the procedures that are low volume in army system) a much lower % of people would get out.		
18	Sep 29 2020 05:31 PM	I think for the initial medical school/HPSP tradeoff, joining the army for financial considerations was worth it. However, for fellowship, given the constraints on military medicine and especially my field, I would not recommend an Army education in fellowship. Additionally, I would highly recommend the individual join the Reserves. With MC officers being MAP'd and with DHA, the tradeoff is no longer worth it.		
19	Sep 29 2020 04:15 PM	Use the Army as much as it will use you. Learn everything offered and take it with you into Civilian life.		
20	Sep 29 2020 03:42 PM	It is important that you understand the needs of the Army/military will always supercede your wants/needs. They will accomodate if possible but understanding this principle will mitigate a lot of frustration down the road		
21	Sep 29 2020 03:34 PM	I think that I would have difficulty giving advice since I think that the future of Army Medicine is uncertain. Many of the changes spearheaded by the DHA have increased the frustrations associated with military medicine. It is unclear that USUHS will continue to exist. Military GME seems to be similarly under assault. However, I have a great job with fantastic quality of life. I think it is a gamble		
22	Sep 29 2020 03:34 PM	Be aware of all aspects of military service. In today's military there is more risk that you may not be able to serve directly in fields of interest. You sign up to serve in the capacity that is best for the Army, and that may mean there is a greater need for your service in a field that is less desirable to you. Additionally, many positions require additional duties that feel that they take you away from your clinical practice. That is the same for any Army officer, but is harder to hear for physicians who go into medicine to be clinicians, but are often tasked to sit on the pharmacy committee, risk management committee, or departmental leadership positions.		
23	Sep 29 2020 01:19 PM	Don't take the money. Join if you want to serve. If you join for the money you'll be a miserable indentured service.		
24	Sep 29 2020 11:24 AM	Know that the Army does not care about any of your personal or family goals while you are serving		
25	Sep 29 2020 09:57 AM	Avoid at all costs. Financial debt is preferable to the life/time debt inflicted by your ADSO.		
26	Sep 29 2020 09:12 AM	Never do it it is not worth it. The Army is completely unwilling to hire civilian providers at reasonable rates, and instead chooses to burn out its active duty providers for minimal pay.		
27	Sep 29 2020 09:03 AM	I think it's a great opportunity depending on your future profession in medicine. I think in surgical sub-specialties, it may not be as appealing after hearing about civilian colleagues first pay checks and opportunities for loan forgiveness with certain contracts.		
28	Sep 29 2020 04:56 AM	DON'T FUCKING DO IT		
29	Sep 29 2020 02:13 AM	Make sure you consider the costs and benefits. There are other ways to pay off debt, such as hospital/practice contracts. My Army career has been good, but there are serious down-sides that I was not aware of before I joined. I probably would have made the same choice, though, all things considered.		
30	Sep 28 2020 09:40 PM	Know what you are getting yourself into. Talk to as many Army Physicians as possible. There is a cost to being a Medical Corps Officer, but I love what I do, who I do it with and who I do it for.		
31	Sep 28 2020 05:57 PM	Don't do it. Take on the debt and live like a resident for the first few years post-residency and it will be paid off and you won't ever have to deal with the Military B.S.		
32	Sep 28 2020 05:41 PM	Go for it		
33	Sep 28 2020 05:30 PM	Don't do it for the money. Hard to predict the effect on family until it happens.		
34	Sep 28 2020 05:23 PM	It is a personal choice based upon where the person is at in their life. If young and single, likely recommend against. If older and with family, consider it since it will make the time period while training more possible		
35	Sep 28 2020 04:58 PM	You must choose what matters most to you. If debt is a concerning stressor and you don't mind serving, you should do it. If you can't stand military service, take the debt.		
36	Sep 24 2020 01:38 PM	Join only if you want to be in the military, not to avoid debt		
37	Sep 20 2020 04:29 AM	Don't. Unless your goal is combat medicine, don't.		
38	Sep 19 2020 02:49 PM	great leadership experience. you get to be more than just a doctor		
39	Sep 17 2020 09:37 PM	Don’t.		
40	Sep 17 2020 09:32 PM	The only reason to consider joining the military to pay for medical school is if you have an overwhelming desire to serve in the military. The numbers CLEARLY favor taking the debt and remaining a civilian.		
41	Sep 16 2020 07:35 PM	Applicants entering medical school have a particular specialty in mind initially. If you were doing a specialty that pays comparable to civilian counterparts the financial benefit is essentially the same. For specialties with much higher civilian counter part pay (IE Otolaryngology, anesthesiology, neurosurgery etc) then there is typically significant financial loss in the long run. Applicant should balance HPSP decision based on long term career goals in medicine.		
42	Sep 16 2020 06:53 PM	Your specialty of choice should play a huge part in making that decision.		
43	Sep 16 2020 01:43 PM	Don’t bother. Medicine is being overrun by incompetent midlevels and big business. Patients are screwed. Go to law school.		
44	Sep 16 2020 01:35 PM	Your joining the army, so be prepared to do a lot army activities like going to field and probably deploying		
45	Sep 16 2020 09:36 AM	Don't do it. You'll come out behind and not worth the b.s.		
46	Sep 16 2020 08:49 AM	Consider the lost opportunity cost/potential earnings lost in addition to location instability with multiple moves while serving on active duty.		
47	Sep 15 2020 05:14 PM	Army medicine is a sinking ship and a setup for demoralization. I say this as someone with time in the military before medical school. I would do almost anything to leave the military right now.		
48	Sep 14 2020 10:11 AM	The military healthcare system is in a period of uncertainty for the following reasons: force reshaping, possible loss of 18000 medical billets across all services, DHA takeover, USUHS may be closed. You may not be able to go into a specialty of your choice because of the force reshaping. You should be ready to pass the new PT test, the ACFT. You should be prepared to deploy.		
49	Sep 11 2020 07:19 PM	Only do it if serving in the Army is of primary importance to you. Understand beforehand that the priorities foisted on you at work may be very different from normal physicians, to include duties that may not be related to healthcare at all. Do not expect to be at the forefront of clinical medicine or research.		
50	Sep 10 2020 08:53 AM	It's a great option and a great training opportunity.		
51	Sep 09 2020 03:57 PM	I would tell them to consider and know what ALL their options are		
52	Sep 09 2020 03:32 PM	probably I would say as long as they don't mind poor leadership and hipocricy, go for it		
53	Sep 09 2020 02:17 PM	You will gain great clinical and supervisory experience at a young age in comparison to your peers and have opportunities to travel the world practicing medicine.		
54	Sep 09 2020 11:16 AM	DHA is a disaster so theu should stay away		
55	Sep 08 2020 01:41 PM	Join the air force instead		
56	Sep 08 2020 01:18 PM	Take into account your own personal situation before making the decision and be careful to know the consequences of the decision, such as different residency option and deployment needs.		
57	Sep 08 2020 01:07 PM	I do not recommend		
58	Sep 08 2020 10:16 AM	Do not do it unless you know you want to be a general surgery, or primary care		
59	Sep 08 2020 08:56 AM	Do it because you want to serve your country, not for finances		
60	Sep 08 2020 08:07 AM	Do not do it for financial reasons alone. There is far too much scrifice to make for this commitment.		
61	Sep 08 2020 07:10 AM	Do not join - especially for the field of surgery. The current deployment tempo makes it nearly impossible to maintain clinical skills, let alone a sustainable family life		
62	Sep 06 2020 08:51 AM	Separation from immediate family (spouse, children) is nearly constant, and debilitating to families. We have very little control of our lives.		
63	Sep 06 2020 08:44 AM	mission is very rewarding, understand concept of needs of the Army and surgeons will deploy so frequently to the point it can place significant hardship on personal life.		
64	Sep 06 2020 08:17 AM	Don’t do it!		
65	Sep 05 2020 01:49 PM	Do it for the right reasons. Don’t let your financial situation be the only reason you join.		
66	Sep 05 2020 12:36 PM	Get as many perspectives as possible before making that decision		
67	Sep 05 2020 12:28 PM	Only do it if you want to be in the military. Money isn’t everything.		
68	Sep 05 2020 11:38 AM	Do it, but know what you share getting into.		
69	Sep 05 2020 09:52 AM	If you are joining strictly for financial reasons, it will be the most regretful decision of your life.		
70	Sep 05 2020 08:11 AM	Be aware that you will be a soldier first physician second. If being soley a physician is your main career objective I would reconsider.		
71	Sep 05 2020 01:51 AM	Do not join the military to pay for medical school		
72	Sep 04 2020 11:17 PM	Don't join the military to pay for medical school. You will be miserable. Join the military if you want to joing the military.		
73	Sep 04 2020 08:33 PM	Dont do it. Its not worth the money. The Army's entire strategy for recruitment and retention is to get people to sign up for committments they will regret. For instance, they are happy to offer you a fellowship and the obligation from it but they do not feel any obligation to put you in a job where you can retain the skills you learned in fellowship. Dont do Army medicine. It is a dying institution. The money is not worth the pain. You get more respect as a surgery resident than you do as an Army Physician. The Army cares absolutely nothing about your ability to maintain your skills or find a job after the military. Dont do it.		
74	Sep 04 2020 07:27 PM	If you are considering going into general or orthopedic surgery, simply don't do it. You will be deployed frequently, and will operate 1/3 to 1/8 as much as your civilian counterparts.		
Q12. If you deployed today, how would your performance at trauma care on day one compare to a civilian physician of the same specialty who is based in a reasonably busy trauma center?				
Answer Choices	Responses			
I would perform much better than a civilian counterpart.	6.38%	6		
I would perform better than a civilian counterpart.	17.02%	16		
I would perform at a similar level to a civilian counterpart.	26.60%	25		
I would perform worse than a civilian counterpart.	22.34%	21		
I would perform much worse than a civilian counterpart.	20.21%	19		
I would not directly or indirectly treat trauma patients on a deployment. Update 9/9/2020: Please skip questions 13-17 if selecting this answer choice.	7.45%	7		
	Answered	94		
	Skipped	0		
Q13. If a typical military physician were deployed today, how would you expect that military physician's trauma care on day one of deployment to compare to a civilian physician of the same specialty based in a reasonably busy trauma center?				
Answer Choices	Responses			
The typical military physician would perform much better than a civilian counterpart.	7.87%	7		
The typical military physician would perform better than a civilian counterpart.	16.85%	15		
The typical military physician would perform at a similar level to a civilian counterpart.	22.47%	20		
The typical military physician would perform worse than a civilian counterpart.	33.71%	30		
The typical military physician would perform much worse than a civilian counterpart.	19.10%	17		
	Answered	89		
	Skipped	5		
Q14. If you deployed today, how would the Army's impact on your skill maintenance affect outcomes of wounded soldiers?				
Answer Choices	Responses			
My Army positions have further developed my critical trauma skills and greatly decreased the risk of harm to soldiers.	5.68%	5		
My Army positions have further developed my critical trauma skills and decreased the risk of harm to soldiers.	4.55%	4		
My Army positions have maintained my ability to perform critical trauma skills without any increase in risk to soldiers.	26.14%	23		
My Army positions have deteriorated my ability to perform critical trauma skills and increased risk of harm to soldiers.	50.00%	44		
My Army positions have deteriorated my ability to perform critical trauma skills and greatly increased the risk of harm to soldiers.	13.64%	12		
	Answered	88		
	Skipped	6		
Q15. How would you rate your clinical skills as they relate to trauma patients against a civilian physician in your specialty?				
Answer Choices	Responses			
My trauma skills are much stronger than a comparable civilian physician.	8.99%	8		
My trauma skills are stronger than a comparable civilian physician.	22.47%	20		
My trauma skills are similar or equal to a comparable civilian physician.	33.71%	30		
My trauma skills are weaker than a comparable civilian physician.	21.35%	19		
My trauma skills are much weaker than a comparable civilian physician.	13.48%	12		
	Answered	89		
	Skipped	5		
Q16. How important is it to you to work in a clinical setting where you can care for a higher-than average percentage of complex trauma patients?				
Answer Choices	Responses			
Very important	28.09%	25		
Moderately important	17.98%	16		
Somewhat important	19.10%	17		
Not important at all	34.83%	31		
I prefer a non-clinical work setting	0.00%	0		
	Answered	89		
	Skipped	5		
Q17. What impact have your Army positions had on your ability to treat complex trauma patients?				
Answer Choices	Responses			
A very positive impact	5.62%	5		
A positive impact	17.98%	16		
Little to no impact either way	32.58%	29		
A negative impact	25.84%	23		
A very negative impact	17.98%	16		
	Answered	89		
	Skipped	5		
Q18. How important is it to you to work in a clinical setting where you can care for a higher-than average percentage of complex medical patients?				
Answer Choices	Responses			
Very important	42.55%	40		
Moderately important	23.40%	22		
Somewhat important	20.21%	19		
Not important at all	12.77%	12		
I prefer a non-clinical work setting. Update 9/9/2020: Please skip questions 19-27 if selecting this answer choice.	1.06%	1		
	Answered	94		
	Skipped	0		
Q19. What impact have your Army positions had on your ability to treat complex medical patients?				
Answer Choices	Responses			
A very positive impact	4.30%	4		
A positive impact	12.90%	12		
Little to no impact either way	17.20%	16		
A negative impact	37.63%	35		
A very negative impact	27.96%	26		
	Answered	93		
	Skipped	1		
Q20. Compared to a number that you feel would be adequate to maintain your desired clinical skills, how would you describe the number of opportunities to perform the relevant procedures in your Army duty positions? Please disregard continuing medical education and off-duty employment extraneous to reserved training time.				
Answer Choices	Responses			
Many more opportunities than would be adequate	2.15%	2		
More opportunities than would be adequate	7.53%	7		
Just enough opportunities	12.90%	12		
Fewer opportunities than would be adequate	30.11%	28		
Far fewer opportunities than would be adequate	43.01%	40		
I do not perform procedures as part of my typical skill set. Update 9/9/2020: Added answer choice.	4.30%	4		
	Answered	93		
	Skipped	1		
Q21. Compared to a number that you feel would be adequate to maintain your desired clinical skills, how would you describe the number of opportunities to perform relevant patient care in your Army duty positions? Please disregard continuing medical education and off-duty employment extraneous to reserved training time.				
Answer Choices	Responses			
Many more opportunities than would be adequate	3.23%	3		
More opportunities than would be adequate	8.60%	8		
Just enough opportunities	27.96%	26		
Fewer opportunities than would be adequate	34.41%	32		
Far fewer opportunities than would be adequate	25.81%	24		
	Answered	93		
	Skipped	1		
Q22. To what extent does local leadership understand the benefit of continuing medical education and/or off-duty employment that you may need to maintain your skills?				
Answer Choices	Responses			
They show strong understanding.	9.68%	9		
They show moderate understanding.	19.35%	18		
They show somewhat understanding.	32.26%	30		
They show little to no understanding.	38.71%	36		
	Answered	93		
	Skipped	1		
Q23. To what extent does senior leadership understand the benefit of continuing medical education and/or off-duty employment that you may need to maintain your skills?				
Answer Choices	Responses			
They show strong understanding.	5.38%	5		
They show moderate understanding.	13.98%	13		
They show somewhat understanding.	23.66%	22		
They show little to no understanding.	56.99%	53		
	Answered	93		
	Skipped	1		
Q24. What role does local leadership play in your pursuit of continuing medical education and/or off-duty employment that you may need to maintain your skills?				
Answer Choices	Responses			
Local leadership strongly supports these pursuits.	6.45%	6		
Local leadership supports these pursuits.	29.03%	27		
Local leadership neither supports nor discourages these pursuits.	36.56%	34		
Local leadership discourages these pursuits.	15.05%	14		
Local leadership strongly discourages these pursuits.	12.90%	12		
	Answered	93		
	Skipped	1		
Q25. What role does senior leadership play in your pursuit of continuing medical education and/or off-duty employment that you may need to maintain your skills?				
Answer Choices	Responses			
Senior leadership strongly supports these pursuits.	2.15%	2		
Senior leadership supports these pursuits.	16.13%	15		
Senior leadership neither supports nor discourages these pursuits.	47.31%	44		
Senior leadership discourages these pursuits.	20.43%	19		
Senior leadership strongly discourages these pursuits.	13.98%	13		
	Answered	93		
	Skipped	1		
Q26. How do you feel deployment length relates to your typical clinical skills?				
Answer Choices	Responses			
Longer deployments greatly improve my typical clinical skills.	1.09%	1		
Longer deployments improve my typical clinical skills.	0.00%	0		
Deployment length has no significant impact on my typical clinical skills.	8.70%	8		
Longer deployments degrade my typical clinical skills.	36.96%	34		
Longer deployments greatly degrade my typical clinical skills.	53.26%	49		
	Answered	92		
	Skipped	2		
Q27. How have your Army positions impacted your anticipated ability to transition to civilian practice within your specialty after separation?Update 9/9/20: Added "anticipated" and "after separation" to question stem for clarification				
Answer Choices	Responses			
My Army positions have made it much easier to transition to a civilian practice.	3.19%	3		
My Army positions have made it easier to transition to a civilian practice.	5.32%	5		
My Army positions have had no significant impact on my ability to transition to a civilian practice.	27.66%	26		
My Army positions have made it more difficult to transition to a civilian practice.	46.81%	44		
My Army positions have made it much more difficult to transition to a civilian practice.	17.02%	16		
	Answered	94		
	Skipped	0		
Q28. ...overall job satisfaction?				
Answer Choices	Responses			
Active duty service provides much higher job satisfaction compared to a civilian career.	4.26%	4		
Active duty service provides higher job satisfaction compared to a civilian career.	7.45%	7		
Active duty service provides about the same level of job satisfaction compared to a civilian career.	28.72%	27		
Active duty service provides lower job satisfaction compared to a civilian career.	35.11%	33		
Active duty service provides much lower job satisfaction compared to a civilian career.	24.47%	23		
	Answered	94		
	Skipped	0		
Q29. ...choice of work hours?				
Answer Choices	Responses			
Active duty service provides much greater choice of work hours compared to a civilian career.	1.06%	1		
Active duty service provides greater choice in work hours compared to a civilian career.	15.96%	15		
Active duty service provides about the same choice in work hours compared to a civilian career.	27.66%	26		
Active duty service provides less choice in work hours compared to a civilian career.	23.40%	22		
Active duty service provides much less choice in work hours compared to a civilian career.	31.91%	30		
	Answered	94		
	Skipped	0		
Q30. ...choice in the nature of your work?				
Answer Choices	Responses			
Active duty service provides much greater choice in the nature of my work compared to a civilian career.	4.26%	4		
Active duty service provides greater choice in the nature of my work compared to a civilian career.	9.57%	9		
Active duty service provides about the same choice in the nature of my work compared to a civilian career.	10.64%	10		
Active duty service provides less choice in the nature of my work compared to a civilian career.	31.91%	30		
Active duty service provides much less choice in the nature of my work compared to a civilian career.	43.62%	41		
	Answered	94		
	Skipped	0		
Q31. ...choice in where you live?				
Answer Choices	Responses			
Active duty service provides much greater choice in where you live compared to a civilian career.	0.00%	0		
Active duty service provides greater choice in where you live compared to a civilian career.	2.13%	2		
Active duty service provides about the same choice in where you live compared to a civilian career.	4.26%	4		
Active duty service provides less choice in where you live compared to a civilian career.	15.96%	15		
Active duty service provides much less choice in where you live compared to a civilian career.	77.66%	73		
	Answered	94		
	Skipped	0		
Q32. ...freedom to practice the kind of medicine you feel passionate about?				
Answer Choices	Responses			
Active duty service provides much greater freedom compared to a civilian career.	6.38%	6		
Active duty service provides greater freedom compared to a civilian career.	5.32%	5		
Active duty service provides about the same freedom compared to a civilian career.	15.96%	15		
Active duty service provides less freedom compared to a civilian career.	32.98%	31		
Active duty service provides much less freedom compared to a civilian career.	39.36%	37		
	Answered	94		
	Skipped	0		
Q33. ...opportunities to attend important medical conferences?				
Answer Choices	Responses			
Active duty service provides many more opportunities compared to a civilian career.	0.00%	0		
Active duty service provides more opportunities compared to a civilian career.	5.32%	5		
Active duty service provides about the same opportunities compared to a civilian career.	19.15%	18		
Active duty service provides fewer opportunities compared to a civilian career.	37.23%	35		
Active duty service provides far fewer opportunities compared to a civilian career.	38.30%	36		
	Answered	94		
	Skipped	0		
Q34. ...opportunities to travel?				
Answer Choices	Responses			
Active duty service provides many more opportunities compared to a civilian career.	7.45%	7		
Active duty service provides more opportunities compared to a civilian career.	20.21%	19		
Active duty service provides about the same opportunities compared to a civilian career.	22.34%	21		
Active duty service provides fewer opportunities compared to a civilian career.	20.21%	19		
Active duty service provides far fewer opportunities compared to a civilian career.	29.79%	28		
	Answered	94		
	Skipped	0		
Q35. ...opportunities to treat under-served populations?				
Answer Choices	Responses			
Active duty service provides many more opportunities compared to a civilian career.	1.06%	1		
Active duty service provides more opportunities compared to a civilian career.	4.26%	4		
Active duty service provides about the same opportunities compared to a civilian career.	14.89%	14		
Active duty service provides fewer opportunities compared to a civilian career.	40.43%	38		
Active duty service provides far fewer opportunities compared to a civilian career.	39.36%	37		
	Answered	94		
	Skipped	0		
Q36. How effectively does active duty service utilize your skills to meet your personal goal of serving your country?				
Answer Choices	Responses			
Very effectively	13.83%	13		
Moderately effectively	19.15%	18		
Somewhat effectively	27.66%	26		
Somewhat ineffectively	9.57%	9		
Ineffectively	12.77%	12		
Very ineffectively	14.89%	14		
Serving my country is not a personal goal of mine.	2.13%	2		
	Answered	94		
	Skipped	0		
Q37. Overall, when comparing your service on active duty against a civilian career path of your choice, how would you respond about pay and benefits?				
Answer Choices	Responses			
Active duty service provides much better pay and benefits compared to a civilian career.	0.00%	0		
Active duty service provides better pay and benefits compared to a civilian career.	5.32%	5		
Active duty service provides about the same pay and benefits compared to a civilian career.	12.77%	12		
Active duty service provides worse pay and benefits compared to a civilian career.	44.68%	42		
Active duty service provides much worse pay and benefits compared to a civilian career.	37.23%	35		
	Answered	94		
	Skipped	0		
Q38. Overall, when comparing your service on active duty to an alternative career as a civilian physician, how would you respond about your ability to provide for the specific needs of your family?				
Answer Choices	Responses			
Active duty service provides a much greater ability to provide for my family's needs compared to a civilian career.	1.06%	1		
Active duty service provides a greater ability to provide for my family's needs compared to a civilian career.	8.51%	8		
Active duty service provides about the same ability to provide for my family's needs compared to a civilian career.	32.98%	31		
Active duty service provides a worse ability to provide for my family's needs compared to a civilian career.	31.91%	30		
Active duty service provides a much worse ability to provide for my family's needs compared to a civilian career.	25.53%	24		
	Answered	94		
	Skipped	0		
Q39. On the current trajectory, how do you expect active duty physician satisfaction will change over the next 5 years?				
Answer Choices	Responses			
Active duty physician satisfaction will improve greatly.	0.00%	0		
Active duty physician satisfaction will improve .	0.00%	0		
Active duty physician satisfaction will not change considerably.	12.77%	12		
Active duty physician satisfaction will worsen .	40.43%	38		
Active duty physician satisfaction will worsen greatly.	46.81%	44		
	Answered	94		
	Skipped	0		
Q40. If your military pay was equal to your expected civilian pay, which statement best describes your feelings regarding extending your service past your active duty service obligation?				
Answer Choices	Responses			
This change alone would likely be sufficient to continue my service past my current obligation.	12.77%	12		
This change alone may be sufficient to continue service past my current obligation.	36.17%	34		
This change alone would likely be insufficient for me to continue service past my current obligation.	19.15%	18		
I would not stay on active duty past my obligation regardless of these pay changes.	26.60%	25		
I plan to stay on active duty past my obligation regardless of these pay changes.	5.32%	5		
	Answered	94		
	Skipped	0		
Q41. Senior Army medical leadership positions are held by individuals who are promoted and assigned to positions based on their leadership skills and administrative training.				
Answer Choices	Responses			
Strongly agree	4.26%	4		
Agree	14.89%	14		
Neither agree nor disagree	22.34%	21		
Disagree	30.85%	29		
Strongly disagree	27.66%	26		
	Answered	94		
	Skipped	0		
Q42. How would you compare Army medical leaders to their corresponding civilian counterparts in leadership and administrative guidance?				
Answer Choices	Responses			
Army medical leaders provide far superior leadership and administrative guidance than corresponding leaders in civilian organizations.	2.15%	2		
Army medical leaders provide superior leadership and administrative guidance than corresponding leaders in civilian organizations.	3.23%	3		
Army medical leaders provide equivalent leadership and administrative guidance than corresponding leaders in civilian organizations.	35.48%	33		
Army medical leaders provide inferior leadership and administrative guidance than corresponding leaders in civilian organizations.	44.09%	41		
Army medical leaders provide far inferior leadership and administrative guidance than corresponding leaders in civilian organizations.	15.05%	14		
	Answered	93		
	Skipped	1		
Q43. How strong of an understanding do you feel Army medical leadership possesses regarding the needs of active duty physicians?				
Answer Choices	Responses			
Strong understanding	1.06%	1		
Moderate understanding	14.89%	14		
Weak understanding	52.13%	49		
Very little to no understanding	31.91%	30		
	Answered	94		
	Skipped	0		
Q44. How would you rate the performance of Army medical leadership in advancing the clinical skills of active duty physicians?				
Answer Choices	Responses			
Army medical leadership performs very well in this area.	0.00%	0		
Army medical leadership performs well in this area.	5.32%	5		
Army medical leadership performs adequately in this area.	19.15%	18		
Army medical leadership performs poorly in this area.	39.36%	37		
Army medical leadership performs very poorly in this area.	36.17%	34		
	Answered	94		
	Skipped	0		
Q45. How would you rate the performance of Army medical leadership in advancing the deployment readiness of active duty physicians?				
Answer Choices	Responses			
Army medical leadership performs very well in this area.	0.00%	0		
Army medical leadership performs well in this area.	9.57%	9		
Army medical leadership performs adequately in this area.	31.91%	30		
Army medical leadership performs poorly in this area.	32.98%	31		
Army medical leadership performs very poorly in this area.	25.53%	24		
	Answered	94		
	Skipped	0		
Q46. How would you rate the performance of Army medical leadership in advancing the job satisfaction and wellness of active duty physicians?				
Answer Choices	Responses			
Army medical leadership performs very well in this area.	0.00%	0		
Army medical leadership performs well in this area.	1.06%	1		
Army medical leadership performs adequately in this area.	9.57%	9		
Army medical leadership performs poorly in this area.	36.17%	34		
Army medical leadership performs very poorly in this area.	53.19%	50		
	Answered	94		
	Skipped	0		
Q47. In general, how do you feel Army medical leaders encourage or discourage efforts by physicians to improve Army healthcare?				
Answer Choices	Responses			
Strongly encourages and/or facilitates	1.06%	1		
Encourages and/or facilitates	10.64%	10		
Neither encourages nor discourages such efforts	31.91%	30		
Discourages and/or hinders	41.49%	39		
Strongly discourages and/or hinders	14.89%	14		
	Answered	94		
	Skipped	0		
Q48. To what extent do you feel encouraged to raise any concerns to your leadership?				
Answer Choices	Responses			
I feel very encouraged to raise concerns to my leadership.	3.19%	3		
I feel encouraged to raise concerns to my leadership.	17.02%	16		
I feel neither encouraged nor discouraged from raising concerns to my leadership.	32.98%	31		
I feel discouraged from raising concerns to my leadership.	30.85%	29		
I feel very discouraged from raising concerns to my leadership.	15.96%	15		
	Answered	94		
	Skipped	0		
Q49. To what extent do you feel encouraged to offer solutions to your leadership?				
Answer Choices	Responses			
I feel very encouraged to offer solutions to my leadership.	3.19%	3		
I feel encouraged to offer solutions to my leadership.	19.15%	18		
I feel neither encouraged nor discouraged from offering solutions to my leadership.	32.98%	31		
I feel discouraged from offering solutions to my leadership.	31.91%	30		
I feel very discouraged from offering solutions to my leadership.	12.77%	12		
	Answered	94		
	Skipped	0		
Q50. How likely is it that the concerns you raise will be adequately addressed?				
Answer Choices	Responses			
My concerns are very likely to be adequately addressed.	1.06%	1		
My concerns are likely to be adequately addressed.	5.32%	5		
My concerns are neither likely nor unlikely to be adequately addressed.	19.15%	18		
My concerns are unlikely to be adequately addressed.	37.23%	35		
My concerns are very unlikely to be adequately addressed.	37.23%	35		
	Answered	94		
	Skipped	0		
Q51. How empowered do you feel to enact change in your organization?				
Answer Choices	Responses			
Very empowered	3.19%	3		
Moderately empowered	13.83%	13		
Somewhat empowered	28.72%	27		
I do not feel empowered at all to enact any change.	54.26%	51		
	Answered	94		
	Skipped	0		
Q52. What is your assessment of the personal risk of retribution due to speaking out against decisions and policies that you disagree with?				
Answer Choices	Responses			
Significant personal risk	22.34%	21		
Moderate personal risk	18.09%	17		
Slight personal risk	32.98%	31		
Little to no risk	26.60%	25		
	Answered	94		
	Skipped	0		
Q53. If you feel there is risk associated with the previous question (question 51), please indicate the forms of retribution that a physician may fear encountering. Choose all that apply.				
Answer Choices	Responses			
Uniformed Code of Military Justice action	15.28%	11		
Additional work duties and/or withdrawal of current support at their current assignment	63.89%	46		
Involuntary extension of military service	5.56%	4		
Medical license and/or credentialing implications	26.39%	19		
Post-military career implications	29.17%	21		
Loss of command position	20.83%	15		
Lower chance of promotion	47.22%	34		
Lower chance of favorable assignments and/or duty stations	69.44%	50		
Out-of-turn deployment and/or assigned task away from their current assignment	50.00%	36		
Other (please specify)	16.67%	12		
	Answered	72		
	Skipped	22		
Respondents	Response Date	Other (please specify)	Tags	
1	Oct 26 2020 08:22 AM	N/A		
2	Oct 09 2020 04:11 AM	i feel it is likely that personal reprisals would take place in a manner that would be difficult to prove legally		
3	Oct 01 2020 10:11 AM	Revoking of permission for off duty employment		
4	Sep 29 2020 05:31 PM	None		
5	Sep 29 2020 03:42 PM	You risk being labeled as a none team player and passed over for positions of responsibility/leadership		
6	Sep 28 2020 09:40 PM	Poor evaluation		
7	Sep 28 2020 05:41 PM	na		
8	Sep 19 2020 02:49 PM	more likely to be singled out during career especially if you’re a minority		
9	Sep 16 2020 09:18 AM	Being placed on a list of "problem" officers		
10	Sep 06 2020 08:44 AM	poor OER/officer evaluation, poor reputation		
11	Sep 05 2020 08:19 AM	Loss of moonlighting ([off-duty employment]) privileges		
12	Sep 04 2020 07:27 PM	Removal of [off-duty employment] approval		
Q54. In general, the reliable replacement of physicians leaving active duty by new graduates with active duty service obligations encourages leadership to work to improve the active duty physician experience.				
Answer Choices	Responses			
Strongly agree	1.08%	1		
Agree	5.38%	5		
Neither agree nor disagree	11.83%	11		
Disagree	40.86%	38		
Strongly disagree	40.86%	38		
	Answered	93		
	Skipped	1		
Q55. In general, the reliable replacement of physicians leaving active duty by new graduates with active duty service obligations discourages leadership from working to improve the active duty physician experience.				
Answer Choices	Responses			
Strongly agree	37.63%	35		
Agree	39.78%	37		
Neither agree nor disagree	16.13%	15		
Disagree	4.30%	4		
Strongly disagree	2.15%	2		
	Answered	93		
	Skipped	1		
Q56. In general, limiting recruitment to attending physicians instead of aspiring medical students would most likely encourage leadership to work to improve the active duty physician experience.				
Answer Choices	Responses			
Strongly agree	23.66%	22		
Agree	37.63%	35		
Neither agree nor disagree	24.73%	23		
Disagree	6.45%	6		
Strongly disagree	7.53%	7		
	Answered	93		
	Skipped	1		
Q57. In general, limiting recruitment to attending physicians instead of aspiring medical students would most likely discourage leadership from working to improve the active duty physician experience.				
Answer Choices	Responses			
Strongly agree	2.17%	2		
Agree	2.17%	2		
Neither agree nor disagree	38.04%	35		
Disagree	33.70%	31		
Strongly disagree	23.91%	22		
	Answered	92		
	Skipped	2		
Q58. Have you seen a psychiatrist, psychotherapist, or other mental health specialists since joining the Army?				
Answer Choices	Responses			
Yes	38.30%	36		
No	61.70%	58		
	Answered	94		
	Skipped	0		
Q59. Are you on medications to control anxiety and/or depression?				
Answer Choices	Responses			
Yes, I started medicating during my active duty service and continue to do so.	13.98%	13		
Yes, I started medicating before my active duty service and continue to do so.	0.00%	0		
No, I have not taken any while on active duty.	78.49%	73		
No, but I have taken them while on active duty	7.53%	7		
	Answered	93		
	Skipped	1		
Q60. How much support do you expect an active duty physician would receive from local leadership in adapting their practice in order to adequately treat their own potential mental health condition?				
Answer Choices	Responses			
More support than what would be adequate	5.32%	5		
Adequate support	46.81%	44		
Less support than what would be adequate	34.04%	32		
Little to no support	13.83%	13		
	Answered	94		
	Skipped	0		
Q61. How much support do you expect an active duty physician would receive from local leadership in adapting their practice in order to adequately address potential burnout?				
Answer Choices	Responses			
More support than what would be adequate	2.13%	2		
Adequate support	17.02%	16		
Less support than what would be adequate	43.62%	41		
Little to no support	37.23%	35		
	Answered	94		
	Skipped	0		
Q62. What is your assessment of Army medical leadership's position regarding physicians seeking out mental health care services?				
Answer Choices	Responses			
Active duty physicians are implicitly and/or explicitly strongly encouraged to seek care.	6.38%	6		
Active duty physicians are implicitly and/or explicitly encouraged to seek care.	27.66%	26		
Active duty physicians are not implicitly and/or explicitly encouraged or discouraged from seeking care.	43.62%	41		
Active duty physicians are implicitly and/or explicitly discouraged from seeking care.	17.02%	16		
Active duty physicians are implicitly and/or explicitly very discouraged from seeking care.	5.32%	5		
	Answered	94		
	Skipped	0		
Q63. What overall effect has serving on active duty had on your mental health?				
Answer Choices	Responses			
Serving on active duty has very positively impacted my mental health.	2.13%	2		
Serving on active duty has positively impacted my mental health.	5.32%	5		
Serving on active duty has had no significant impact my mental health.	42.55%	40		
Serving on active duty has negatively impacted my mental health.	38.30%	36		
Serving on active duty has very negatively impacted my mental health.	11.70%	11		
	Answered	94		
	Skipped	0		
Q64. How has your own mental health and/or burnout level impacted the quality of care you provide?				
Answer Choices	Responses			
The care I provide has been very positively affected.	1.08%	1		
The care I provide has been positively affected.	2.15%	2		
The care I provide has not been affected.	67.74%	63		
The care I provide has been negatively affected.	25.81%	24		
The care I provide has been very negatively affected.	3.23%	3		
	Answered	93		
	Skipped	1		
Q65. Having little interest or pleasure in doing things				
Answer Choices	Responses			
Nearly every day.	3.19%	3		
More than half the days.	3.19%	3		
Several days.	20.21%	19		
Not at all.	73.40%	69		
	Answered	94		
	Skipped	0		
Q66. Feeling down, depressed, or hopeless				
Answer Choices	Responses			
Nearly every day.	3.19%	3		
More than half the days.	4.26%	4		
Several days.	25.53%	24		
Not at all.	67.02%	63		
	Answered	94		
	Skipped	0		
Q67. Trouble falling or staying asleep, or sleeping too much				
Answer Choices	Responses			
Nearly every day.	3.23%	3		
More than half the days.	7.53%	7		
Several days.	22.58%	21		
Not at all.	66.67%	62		
	Answered	93		
	Skipped	1		
Q68. Feeling tired or having little energy				
Answer Choices	Responses			
Nearly every day.	7.45%	7		
More than half the days.	7.45%	7		
Several days.	41.49%	39		
Not at all.	43.62%	41		
	Answered	94		
	Skipped	0		
Q69. Poor appetite or overeating				
Answer Choices	Responses			
Nearly every day.	2.13%	2		
More than half the days.	4.26%	4		
Several days.	17.02%	16		
Not at all.	76.60%	72		
	Answered	94		
	Skipped	0		
Q70. Feeling bad about yourself - or that you are a failure or have let yourself or your family down				
Answer Choices	Responses			
Nearly every day.	3.19%	3		
More than half the days.	2.13%	2		
Several days.	24.47%	23		
Not at all.	70.21%	66		
	Answered	94		
	Skipped	0		
Q71. Trouble concentrating on things, such as reading the newspaper or watching television				
Answer Choices	Responses			
Nearly every day.	1.08%	1		
More than half the days.	5.38%	5		
Several days.	15.05%	14		
Not at all.	78.49%	73		
	Answered	93		
	Skipped	1		
Q72. Moving or speaking so slowly that other people could have noticed. Or the opposite - being so fidgety or restless that you have been moving around a lot more than usual				
Answer Choices	Responses			
Nearly every day.	0.00%	0		
More than half the days.	2.13%	2		
Several days.	6.38%	6		
Not at all.	91.49%	86		
	Answered	94		
	Skipped	0		
Q73. Thoughts that you would be better off dead, or of hurting yourself				
Answer Choices	Responses			
Nearly every day.	0.00%	0		
More than half the days.	2.13%	2		
Several days.	2.13%	2		
Not at all.	95.74%	90		
	Answered	94		
	Skipped	0		
Q74. If you checked off any problems, how difficult have these problems made it for you to do your work, take care of things at home, or get along with other people?				
Answer Choices	Responses			
Extremely difficult.	1.06%	1		
Very difficult.	0.00%	0		
Somewhat difficult.	29.79%	28		
Not difficult at all.	29.79%	28		
I did not check off any problems.	39.36%	37		
	Answered	94		
	Skipped	0		
Q75. Which of the following best describes your military status?				
Answer Choices	Responses			
Active Duty Army	100.00%	94		
Reserve Army on Active Duty Orders	0.00%	0		
Reserve Army not on Active Duty Orders	0.00%	0		
Retiree	0.00%	0		
Separated but not retired	0.00%	0		
Other (please specify)	0.00%	0		
	Answered	94		
	Skipped	0		
Q76. What is your level of training?				
Answer Choices	Responses			
Medical Student	0.00%	0		
Intern	0.00%	0		
Resident	0.00%	0		
Fellow	3.19%	3		
Attending Physician	96.81%	91		
	Answered	94		
	Skipped	0		
Q77. Did you complete a fellowship?				
Answer Choices	Responses			
No.	64.89%	61		
I am currently in a fellowship program.	2.13%	2		
I have completed a fellowship program through military service (completed while active duty).	26.60%	25		
I have completed a fellowship program, but it was not affiliated with the military.	6.38%	6		
	Answered	94		
	Skipped	0		
Q78. What is your specialty? If you are a general medical officer, please indicate as such.				
Answered	87			
Skipped	7			
Respondents	Response Date	Responses	Tags	
1	Oct 26 2020 08:22 AM	Emergency Medicine		
2	Oct 12 2020 05:34 PM	General Pediatrician		
3	Oct 09 2020 04:11 AM	i would prefer to not provide this informaiton as i fear reprisal for being honest about the quality of army medicine		
4	Oct 07 2020 01:48 PM	Pulmonary/Critical Care		
5	Oct 03 2020 10:53 AM	General Surgeon		
6	Oct 01 2020 03:04 PM	Pulmonary/Critical Care		
7	Oct 01 2020 10:11 AM	Pulmonary / Critical Care / Sleep Medicine		
8	Sep 30 2020 08:27 PM	prefer not to say		
9	Sep 30 2020 06:31 PM	Pediatrics		
10	Sep 30 2020 01:13 PM	Cardiothoracic Surgery		
11	Sep 30 2020 12:11 PM	Cardiology		
12	Sep 30 2020 11:46 AM	OBGYN		
13	Sep 30 2020 11:09 AM	emergency medicine		
14	Sep 30 2020 10:58 AM	Reproductive Endocrinology and Infertility		
15	Sep 30 2020 09:21 AM	Cardiology		
16	Sep 30 2020 09:10 AM	Critical Care Medicine		
17	Sep 30 2020 08:32 AM	IM		
18	Sep 29 2020 09:37 PM	family medicine		
19	Sep 29 2020 05:31 PM	OB/GYN		
20	Sep 29 2020 03:42 PM	child and adolescent psychiatrist		
21	Sep 29 2020 03:34 PM	ObGyn		
22	Sep 29 2020 03:34 PM	Gastroenterology		
23	Sep 29 2020 03:11 PM	fm		
24	Sep 29 2020 01:19 PM	Orthopedic Surgery		
25	Sep 29 2020 11:24 AM	Emergency Medicine		
26	Sep 29 2020 09:12 AM	Psychiatry		
27	Sep 29 2020 09:03 AM	Orthopaedic surgery		
28	Sep 29 2020 08:43 AM	General Surgeon		
29	Sep 29 2020 07:25 AM	Pediatrics		
30	Sep 29 2020 04:56 AM	Internal Medicine		
31	Sep 29 2020 02:13 AM	Family Medicine		
32	Sep 28 2020 09:40 PM	Psychiatry		
33	Sep 28 2020 06:07 PM	Radiology		
34	Sep 28 2020 05:57 PM	Interventional Radiology		
35	Sep 28 2020 05:41 PM	Internal Medicine		
36	Sep 28 2020 05:30 PM	pediatrics		
37	Sep 28 2020 05:23 PM	orthopedics		
38	Sep 28 2020 05:18 PM	REI		
39	Sep 28 2020 05:00 PM	Family Medicine Hospitalist		
40	Sep 28 2020 04:58 PM	Emergency medicine		
41	Sep 24 2020 01:38 PM	Orthopaedic surgeon		
42	Sep 24 2020 11:26 AM	Orthopaedics		
43	Sep 20 2020 04:29 AM	EM		
44	Sep 19 2020 02:49 PM	occupation and environmental medicine (preventive medicine)		
45	Sep 17 2020 09:37 PM	Emergency Medicine		
46	Sep 17 2020 09:32 PM	Pediatrics / GMO		
47	Sep 16 2020 07:35 PM	GMO		
48	Sep 16 2020 06:53 PM	CT surgery		
49	Sep 16 2020 01:43 PM	EM		
50	Sep 16 2020 01:35 PM	Family medicine		
51	Sep 16 2020 09:36 AM	PM&R		
52	Sep 16 2020 09:18 AM	Psychiatry		
53	Sep 16 2020 08:49 AM	Emergency Medicine		
54	Sep 15 2020 05:14 PM	Psychiatry		
55	Sep 14 2020 10:11 AM	Internal Medicine/Rheumatology		
56	Sep 12 2020 01:02 PM	Pediatrics		
57	Sep 11 2020 07:19 PM	Psychiatry		
58	Sep 10 2020 08:53 AM	Family Medicine		
59	Sep 09 2020 07:48 PM	>>>		
60	Sep 09 2020 03:57 PM	Family Medicine, Flight Surgeon		
61	Sep 09 2020 03:32 PM	Emergency Medicine		
62	Sep 09 2020 02:17 PM	psychiatry		
63	Sep 08 2020 01:41 PM	Neonatologist		
64	Sep 08 2020 01:18 PM	Aerospace		
65	Sep 08 2020 01:07 PM	General Surgeon		
66	Sep 08 2020 12:18 PM	General Surgery		
67	Sep 08 2020 10:16 AM	General Surgery		
68	Sep 08 2020 08:56 AM	OB/GYN		
69	Sep 08 2020 08:07 AM	Orthopaedic Surgeon		
70	Sep 08 2020 07:10 AM	General Surgery		
71	Sep 08 2020 03:22 AM	Preventive Medicine		
72	Sep 07 2020 07:49 PM	pediatrics		
73	Sep 06 2020 08:37 PM	Orthopedics		
74	Sep 06 2020 08:51 AM	EM		
75	Sep 06 2020 08:17 AM	EMERGENCY MEDICINE		
76	Sep 05 2020 01:49 PM	Emergency Medicine		
77	Sep 05 2020 12:36 PM	EM		
78	Sep 05 2020 12:28 PM	Emergency Medicine		
79	Sep 05 2020 11:38 AM	EM		
80	Sep 05 2020 09:52 AM	Emergency Medicine		
81	Sep 05 2020 08:19 AM	Emergency Medicine		
82	Sep 05 2020 08:16 AM	Emergency Medicine		
83	Sep 05 2020 08:11 AM	FM		
84	Sep 05 2020 07:12 AM	Emergency Medicine		
85	Sep 05 2020 01:51 AM	Cardiothoracic Surgery		
86	Sep 04 2020 11:17 PM	EM		
87	Sep 04 2020 07:27 PM	Orthopedic Surgery		
Q79. Which Army program did you take part in?				
Answer Choices	Responses			
Health Professions Scholarship Program	76.60%	72		
Uniformed Services University of Health Science	20.21%	19		
Healthcare Professions Loan Repayment Program	1.06%	1		
None of the above.	2.13%	2		
	Answered	94		
	Skipped	0		
Q80. What was your prior military service experience (before medical training)?				
Answer Choices	Responses			
No prior military service experience.	61.29%	57		
ROTC or military academy	27.96%	26		
Prior active duty enlisted experience	7.53%	7		
Prior active duty officer experience	8.60%	8		
Prior reserve enlisted experience	2.15%	2		
Prior reserve officer experience	1.08%	1		
Other	1.08%	1		
	Answered	93		
	Skipped	1		
Q81. In years, how long have you been serving on active duty (including post-graduate training)?				
Answer Choices	Average Number	Total Number	Responses	
Years served on active duty	10.4787234	985	100.00%	94
			Answered	94
			Skipped	0
Respondents	Response Date		Tags	
1	Oct 26 2020 08:22 AM	6		
2	Oct 12 2020 05:34 PM	11		
3	Oct 09 2020 04:11 AM	9		
4	Oct 07 2020 01:48 PM	10		
5	Oct 03 2020 10:53 AM	11		
6	Oct 01 2020 03:31 PM	8		
7	Oct 01 2020 03:04 PM	7		
8	Oct 01 2020 10:11 AM	10		
9	Sep 30 2020 08:27 PM	10		
10	Sep 30 2020 06:31 PM	13		
11	Sep 30 2020 01:13 PM	14		
12	Sep 30 2020 12:11 PM	10		
13	Sep 30 2020 11:46 AM	13		
14	Sep 30 2020 11:09 AM	5		
15	Sep 30 2020 10:58 AM	12		
16	Sep 30 2020 09:21 AM	20		
17	Sep 30 2020 09:10 AM	16		
18	Sep 30 2020 08:32 AM	5		
19	Sep 29 2020 09:37 PM	4		
20	Sep 29 2020 05:31 PM	12		
21	Sep 29 2020 04:15 PM	12		
22	Sep 29 2020 03:42 PM	16		
23	Sep 29 2020 03:34 PM	9		
24	Sep 29 2020 03:34 PM	19		
25	Sep 29 2020 03:11 PM	6		
26	Sep 29 2020 01:19 PM	10		
27	Sep 29 2020 11:24 AM	17		
28	Sep 29 2020 09:57 AM	19		
29	Sep 29 2020 09:12 AM	9		
30	Sep 29 2020 09:03 AM	5		
31	Sep 29 2020 08:43 AM	15		
32	Sep 29 2020 07:25 AM	4		
33	Sep 29 2020 04:56 AM	6		
34	Sep 29 2020 02:13 AM	4		
35	Sep 28 2020 09:40 PM	24		
36	Sep 28 2020 06:07 PM	13		
37	Sep 28 2020 05:57 PM	9		
38	Sep 28 2020 05:41 PM	6		
39	Sep 28 2020 05:30 PM	7		
40	Sep 28 2020 05:23 PM	16		
41	Sep 28 2020 05:18 PM	16		
42	Sep 28 2020 05:00 PM	11		
43	Sep 28 2020 04:58 PM	7		
44	Sep 24 2020 01:38 PM	7		
45	Sep 24 2020 11:26 AM	3		
46	Sep 20 2020 04:29 AM	26		
47	Sep 19 2020 02:49 PM	6		
48	Sep 17 2020 09:37 PM	9		
49	Sep 17 2020 09:32 PM	8		
50	Sep 16 2020 07:35 PM	13		
51	Sep 16 2020 06:53 PM	10		
52	Sep 16 2020 01:43 PM	13		
53	Sep 16 2020 01:35 PM	5		
54	Sep 16 2020 09:36 AM	5		
55	Sep 16 2020 09:18 AM	5		
56	Sep 16 2020 08:49 AM	7		
57	Sep 15 2020 05:14 PM	10		
58	Sep 14 2020 10:11 AM	8		
59	Sep 12 2020 01:02 PM	13		
60	Sep 11 2020 07:19 PM	7		
61	Sep 10 2020 08:53 AM	12		
62	Sep 09 2020 07:48 PM	9		
63	Sep 09 2020 03:57 PM	6		
64	Sep 09 2020 03:32 PM	17		
65	Sep 09 2020 02:17 PM	13		
66	Sep 09 2020 11:16 AM	14		
67	Sep 08 2020 01:41 PM	6		
68	Sep 08 2020 01:18 PM	13		
69	Sep 08 2020 01:07 PM	14		
70	Sep 08 2020 12:18 PM	26		
71	Sep 08 2020 11:04 AM	12		
72	Sep 08 2020 10:16 AM	18		
73	Sep 08 2020 08:56 AM	7		
74	Sep 08 2020 08:07 AM	6		
75	Sep 08 2020 07:10 AM	13		
76	Sep 08 2020 03:22 AM	22		
77	Sep 07 2020 07:49 PM	10		
78	Sep 06 2020 08:37 PM	7		
79	Sep 06 2020 08:51 AM	5		
80	Sep 06 2020 08:44 AM	15		
81	Sep 06 2020 08:17 AM	8		
82	Sep 05 2020 01:49 PM	6		
83	Sep 05 2020 12:36 PM	3		
84	Sep 05 2020 12:28 PM	7		
85	Sep 05 2020 11:38 AM	20		
86	Sep 05 2020 09:52 AM	7		
87	Sep 05 2020 08:19 AM	5		
88	Sep 05 2020 08:16 AM	7		
89	Sep 05 2020 08:11 AM	8		
90	Sep 05 2020 07:12 AM	6		
91	Sep 05 2020 01:51 AM	14		
92	Sep 04 2020 11:17 PM	8		
93	Sep 04 2020 08:33 PM	12		
94	Sep 04 2020 07:27 PM	8		
Q82. In years, how long did you serve on active duty prior to starting medical school?				
Answer Choices	Average Number	Total Number	Responses	
Years on active duty prior to medical school	1.98245614	113	100.00%	57
			Answered	57
			Skipped	37
Respondents	Response Date		Tags	
1	Oct 26 2020 08:22 AM	0		
2	Oct 12 2020 05:34 PM	0		
3	Oct 09 2020 04:11 AM	1		
4	Oct 07 2020 01:48 PM	0		
5	Oct 01 2020 03:31 PM	0		
6	Oct 01 2020 10:11 AM	0		
7	Sep 30 2020 06:31 PM	3		
8	Sep 30 2020 11:46 AM	3		
9	Sep 30 2020 11:09 AM	0		
10	Sep 30 2020 09:10 AM	0		
11	Sep 29 2020 09:37 PM	0		
12	Sep 29 2020 04:15 PM	0		
13	Sep 29 2020 03:42 PM	4		
14	Sep 29 2020 03:34 PM	4		
15	Sep 29 2020 03:34 PM	0		
16	Sep 29 2020 11:24 AM	15		
17	Sep 29 2020 09:57 AM	4		
18	Sep 29 2020 07:25 AM	0		
19	Sep 29 2020 04:56 AM	0		
20	Sep 29 2020 02:13 AM	0		
21	Sep 28 2020 09:40 PM	9		
22	Sep 28 2020 06:07 PM	0		
23	Sep 28 2020 05:57 PM	0		
24	Sep 28 2020 05:23 PM	6		
25	Sep 28 2020 05:00 PM	0		
26	Sep 28 2020 04:58 PM	0		
27	Sep 24 2020 01:38 PM	0		
28	Sep 20 2020 04:29 AM	16		
29	Sep 19 2020 02:49 PM	0		
30	Sep 16 2020 07:35 PM	0		
31	Sep 16 2020 06:53 PM	0		
32	Sep 16 2020 09:36 AM	0		
33	Sep 16 2020 08:49 AM	0		
34	Sep 15 2020 05:14 PM	5		
35	Sep 14 2020 10:11 AM	0		
36	Sep 11 2020 07:19 PM	0		
37	Sep 10 2020 08:53 AM	4		
38	Sep 09 2020 03:57 PM	0		
39	Sep 09 2020 03:32 PM	3		
40	Sep 09 2020 11:16 AM	0		
41	Sep 08 2020 01:18 PM	4		
42	Sep 08 2020 01:07 PM	4		
43	Sep 08 2020 12:18 PM	11		
44	Sep 08 2020 10:16 AM	0		
45	Sep 08 2020 07:10 AM	4		
46	Sep 08 2020 03:22 AM	7		
47	Sep 07 2020 07:49 PM	0		
48	Sep 06 2020 08:37 PM	0		
49	Sep 06 2020 08:51 AM	0		
50	Sep 05 2020 01:49 PM	0		
51	Sep 05 2020 12:36 PM	0		
52	Sep 05 2020 11:38 AM	4		
53	Sep 05 2020 08:19 AM	0		
54	Sep 05 2020 08:16 AM	0		
55	Sep 05 2020 08:11 AM	0		
56	Sep 04 2020 08:33 PM	2		
57	Sep 04 2020 07:27 PM	0		
Q83. In years, how long is/was your initial active duty service obligation (beginning immediately after postgraduate education)?				
Answer Choices	Average Number	Total Number	Responses	
Years of initial active duty service obligation	6.11827957	569	100.00%	93
			Answered	93
			Skipped	1
Respondents	Response Date		Tags	
1	Oct 26 2020 08:22 AM	4		
2	Oct 12 2020 05:34 PM	4		
3	Oct 09 2020 04:11 AM	8		
4	Oct 07 2020 01:48 PM	7		
5	Oct 03 2020 10:53 AM	12		
6	Oct 01 2020 03:31 PM	4		
7	Oct 01 2020 03:04 PM	4		
8	Oct 01 2020 10:11 AM	3		
9	Sep 30 2020 08:27 PM	7		
10	Sep 30 2020 06:31 PM	4		
11	Sep 30 2020 01:13 PM	5		
12	Sep 30 2020 12:11 PM	5		
13	Sep 30 2020 11:46 AM	9		
14	Sep 30 2020 11:09 AM	11		
15	Sep 30 2020 10:58 AM	4		
16	Sep 30 2020 09:21 AM	7		
17	Sep 30 2020 09:10 AM	4		
18	Sep 30 2020 08:32 AM	4		
19	Sep 29 2020 09:37 PM	4		
20	Sep 29 2020 05:31 PM	7		
21	Sep 29 2020 04:15 PM	4		
22	Sep 29 2020 03:42 PM	4		
23	Sep 29 2020 03:34 PM	4		
24	Sep 29 2020 03:34 PM	3		
25	Sep 29 2020 03:11 PM	4		
26	Sep 29 2020 01:19 PM	4		
27	Sep 29 2020 11:24 AM	4		
28	Sep 29 2020 09:57 AM	13		
29	Sep 29 2020 09:12 AM	7		
30	Sep 29 2020 09:03 AM	4		
31	Sep 29 2020 08:43 AM	12		
32	Sep 29 2020 07:25 AM	4		
33	Sep 29 2020 04:56 AM	4		
34	Sep 29 2020 02:13 AM	4		
35	Sep 28 2020 09:40 PM	3		
36	Sep 28 2020 06:07 PM	6		
37	Sep 28 2020 05:57 PM	7		
38	Sep 28 2020 05:41 PM	7		
39	Sep 28 2020 05:30 PM	4		
40	Sep 28 2020 05:23 PM	7		
41	Sep 28 2020 05:18 PM	8		
42	Sep 28 2020 05:00 PM	4		
43	Sep 28 2020 04:58 PM	4		
44	Sep 24 2020 01:38 PM	4		
45	Sep 24 2020 11:26 AM	9		
46	Sep 20 2020 04:29 AM	4		
47	Sep 19 2020 02:49 PM	4		
48	Sep 17 2020 09:37 PM	4		
49	Sep 17 2020 09:32 PM	7		
50	Sep 16 2020 07:35 PM	4		
51	Sep 16 2020 06:53 PM	9		
52	Sep 16 2020 01:43 PM	14		
53	Sep 16 2020 01:35 PM	4		
54	Sep 16 2020 09:36 AM	4		
55	Sep 16 2020 09:18 AM	9		
56	Sep 16 2020 08:49 AM	4		
57	Sep 15 2020 05:14 PM	4		
58	Sep 14 2020 10:11 AM	8		
59	Sep 12 2020 01:02 PM	8		
60	Sep 11 2020 07:19 PM	9		
61	Sep 10 2020 08:53 AM	12		
62	Sep 09 2020 07:48 PM	3		
63	Sep 09 2020 03:57 PM	9		
64	Sep 09 2020 03:32 PM	6		
65	Sep 09 2020 02:17 PM	7		
66	Sep 09 2020 11:16 AM	7		
67	Sep 08 2020 01:41 PM	7		
68	Sep 08 2020 01:18 PM	8		
69	Sep 08 2020 01:07 PM	9		
70	Sep 08 2020 12:18 PM	5		
71	Sep 08 2020 11:04 AM	4		
72	Sep 08 2020 10:16 AM	7		
73	Sep 08 2020 08:56 AM	4		
74	Sep 08 2020 07:10 AM	18		
75	Sep 08 2020 03:22 AM	7		
76	Sep 07 2020 07:49 PM	4		
77	Sep 06 2020 08:37 PM	4		
78	Sep 06 2020 08:51 AM	4		
79	Sep 06 2020 08:44 AM	8		
80	Sep 06 2020 08:17 AM	9		
81	Sep 05 2020 01:49 PM	4		
82	Sep 05 2020 12:36 PM	8		
83	Sep 05 2020 12:28 PM	4		
84	Sep 05 2020 11:38 AM	4		
85	Sep 05 2020 09:52 AM	8		
86	Sep 05 2020 08:19 AM	4		
87	Sep 05 2020 08:16 AM	4		
88	Sep 05 2020 08:11 AM	10		
89	Sep 05 2020 07:12 AM	4		
90	Sep 05 2020 01:51 AM	9		
91	Sep 04 2020 11:17 PM	4		
92	Sep 04 2020 08:33 PM	9		
93	Sep 04 2020 07:27 PM	4		
Q84. In years, how long do you plan to spend on active duty in the Army beyond your initial active duty service obligation?				
Answer Choices	Average Number	Total Number	Responses	
Planned years beyond active duty service obligation	5.492307692	357	100.00%	65
			Answered	65
			Skipped	29
Respondents	Response Date		Tags	
1	Oct 26 2020 08:22 AM	20		
2	Oct 12 2020 05:34 PM	16		
3	Oct 07 2020 01:48 PM	0		
4	Oct 01 2020 03:04 PM	4		
5	Oct 01 2020 10:11 AM	0		
6	Sep 30 2020 06:31 PM	20		
7	Sep 30 2020 12:11 PM	1		
8	Sep 30 2020 11:46 AM	1		
9	Sep 30 2020 11:09 AM	0		
10	Sep 30 2020 10:58 AM	11		
11	Sep 30 2020 09:21 AM	7		
12	Sep 30 2020 09:10 AM	16		
13	Sep 29 2020 09:37 PM	0		
14	Sep 29 2020 04:15 PM	14		
15	Sep 29 2020 03:42 PM	6		
16	Sep 29 2020 03:34 PM	16		
17	Sep 29 2020 01:19 PM	0		
18	Sep 29 2020 11:24 AM	0		
19	Sep 29 2020 07:25 AM	0		
20	Sep 29 2020 04:56 AM	0		
21	Sep 29 2020 02:13 AM	0		
22	Sep 28 2020 09:40 PM	12		
23	Sep 28 2020 06:07 PM	0		
24	Sep 28 2020 05:57 PM	0		
25	Sep 28 2020 05:41 PM	8		
26	Sep 28 2020 05:30 PM	4		
27	Sep 28 2020 05:23 PM	3		
28	Sep 28 2020 05:18 PM	20		
29	Sep 28 2020 05:00 PM	1		
30	Sep 28 2020 04:58 PM	4		
31	Sep 24 2020 01:38 PM	0		
32	Sep 20 2020 04:29 AM	0		
33	Sep 19 2020 02:49 PM	20		
34	Sep 16 2020 07:35 PM	4		
35	Sep 16 2020 06:53 PM	5		
36	Sep 16 2020 09:36 AM	0		
37	Sep 16 2020 08:49 AM	0		
38	Sep 14 2020 10:11 AM	12		
39	Sep 12 2020 01:02 PM	12		
40	Sep 11 2020 07:19 PM	0		
41	Sep 10 2020 08:53 AM	21		
42	Sep 09 2020 03:57 PM	0		
43	Sep 09 2020 03:32 PM	5		
44	Sep 09 2020 02:17 PM	9		
45	Sep 09 2020 11:16 AM	0		
46	Sep 08 2020 01:18 PM	0		
47	Sep 08 2020 01:07 PM	0		
48	Sep 08 2020 12:18 PM	1		
49	Sep 08 2020 11:04 AM	16		
50	Sep 08 2020 10:16 AM	0		
51	Sep 08 2020 08:56 AM	4		
52	Sep 08 2020 07:10 AM	0		
53	Sep 08 2020 03:22 AM	13		
54	Sep 06 2020 08:37 PM	0		
55	Sep 06 2020 08:51 AM	0		
56	Sep 06 2020 08:44 AM	20		
57	Sep 05 2020 01:49 PM	0		
58	Sep 05 2020 12:36 PM	0		
59	Sep 05 2020 11:38 AM	20		
60	Sep 05 2020 08:19 AM	0		
61	Sep 05 2020 08:16 AM	1		
62	Sep 05 2020 08:11 AM	0		
63	Sep 05 2020 01:51 AM	5		
64	Sep 04 2020 11:17 PM	5		
65	Sep 04 2020 07:27 PM	0		
Q85. How likely are you to remain on active duty past your current obligation?				
Answer Choices	Responses			
Very Likely	13.83%	13		
Moderately likely	6.38%	6		
Somewhat likely	12.77%	12		
Not at all likely	67.02%	63		
	Answered	94		
	Skipped	0		
